# Solid-state molecular organometallic chemistry (SMOM): a user guide to *in crystallo* single-crystal to single-crystal transformations using solid/gas methods

**DOI:** 10.1039/d6dt00444j

**Published:** 2026-03-26

**Authors:** Kristof M. Altus, Samantha K. Furfari, Joe C. Goodall, Matthew R. Gyton, Jack H. Heaton, Chloe L. Johnson, Alasdair I. McKay, Mads Sondrup Møller, Sebastian D. Pike, Andrew S. Weller

**Affiliations:** a Department of Chemistry, University of York Heslington York YO10 5DD UK andrew.weller@york.ac.uk; b School of Science, University of Wollongong Wollongong NSW 2522 Australia; c School of Chemistry, University of New South Wales Sydney NSW 2052 Australia; d School of Chemistry, Monash University Melbourne Victoria 3800 Australia; e Department of Chemistry, University of Warwick Coventry CV4 7AL UK

## Abstract

*In crystallo* solid-state molecular organometallic chemistry (SMOM) offers unique opportunities in organometallic synthesis and catalysis. The methodology provides routes to synthesise complexes that are challenging to isolate and characterise in solution, with the confined lattice environment providing additional opportunities to control reaction pathways and selectivities in both synthesis and catalysis. The power of the method comes in its simplicity, combined with the variety of analytical techniques that can be used to characterise the products. This tutorial review is a user guide to SMOM, stepping though each aspect that needs to be considered for such solid/gas *in crystallo* reactivity, and encourages others to use the method in their own research.

## Abstract 2

This tutorial review provides a practical guide for solid/gas *in crystallo* solid-state molecular organometallic chemistry (SMOM).

## Graphical abstract

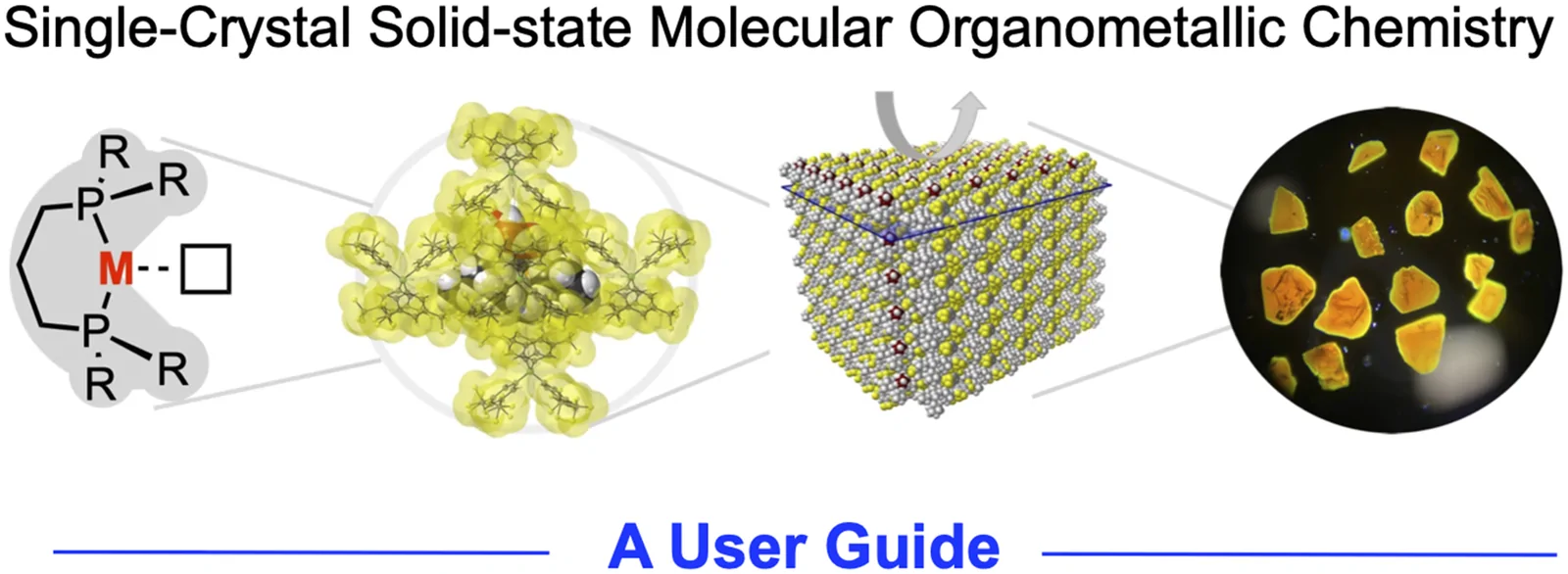

**Key learning points**

(1) An introduction to solid/gas *in crystallo* solid-state molecular organometallic chemistry (SMOM) and a comparison with other solid-state organometallic techniques.

(2) Overview of the design principles of SMOM and general considerations needed to enable successful reactivity *in crystallo*.

(3) A step-by-step user guide to SMOM including: preparation of crystalline material, single-crystal X-ray diffraction, bulk reactivity considerations, gas purity and handling, solid/vapour reactivity and the use of NMR spectroscopy in SMOM.

## 1. Introduction: the SMOM concept

**Scheme 1:**
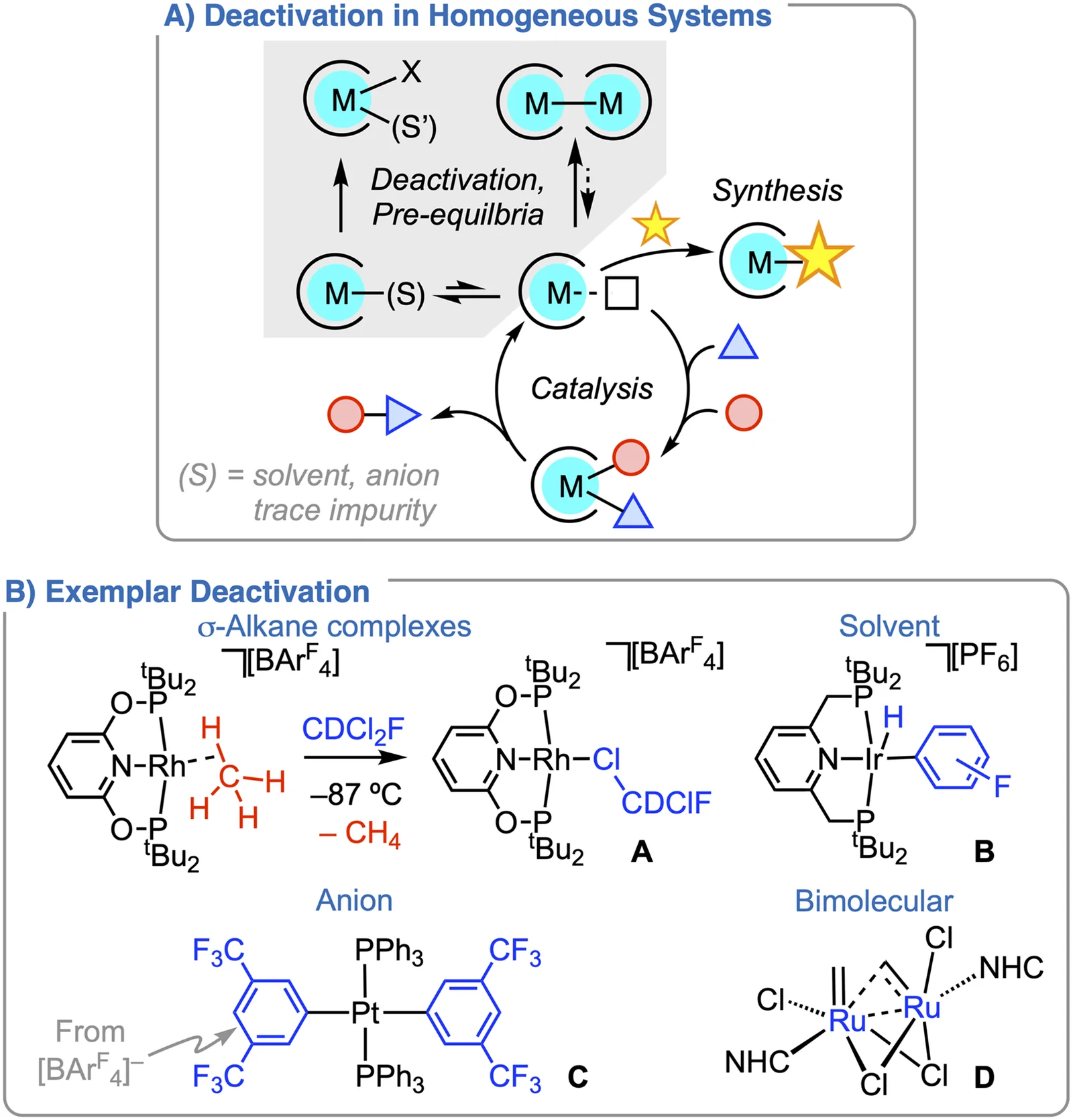
Deactivation pathways (A) and exemplar deactivation products (B) in solution-based organometallic chemistry. Ar^F^ = 3,5-(CF_3_)_2_C_6_H_3_, NHC = N-heterocyclic carbene.

Synthetic and catalytic organometallic chemistry, and molecular chemistry more generally, has traditionally been associated with the use of solution-based methods. By using an appropriate solvent, interactions of the reactants are facilitated, control of reaction conditions and temperature is straightforward, and subsequent purification of product, for example by recrystallisation, is possible.^1^ While convenient, and of course well-established for even very air- and moisture-sensitive systems, the use of solvent can cause problems if it acts in a non-innocent manner. For example, a ligand or catalytic substrate that binds very weakly can be competitively displaced by solvent, establishing potentially undesirable pre-equilibria (Scheme 1A) even if that solvent is not considered nucleophilic. This is because the solvent is often in a significant excess compared with the complex of interest. For example, in a typical NMR-scale experiment, 10 mg of an organometallic complex of *M*_w_ = 1000 g mol^−1^ dissolved in 0.4 cm^3^ of CD_2_Cl_2_ results in a ∼600-fold excess of solvent. An example of this is the displacement of the very weakly binding ligand methane by CDCl_2_F at −87 °C, AScheme 1B.^2^ Further complications can arise from the solvent undergoing an irreversible reaction with the complex of interest (*e.g.* C–halogen,^3,4^ or C–H,^5,6^ activation, B); the decomposition of cationic complexes by reaction with a partnering anion, *e.g.*C;^7–9^ the facile formation of higher nuclearity aggregates in solution, *e.g.*D,^10^ or the reaction with trace, but persistent, impurities present in the solvent.^11^

**Scheme 2:**
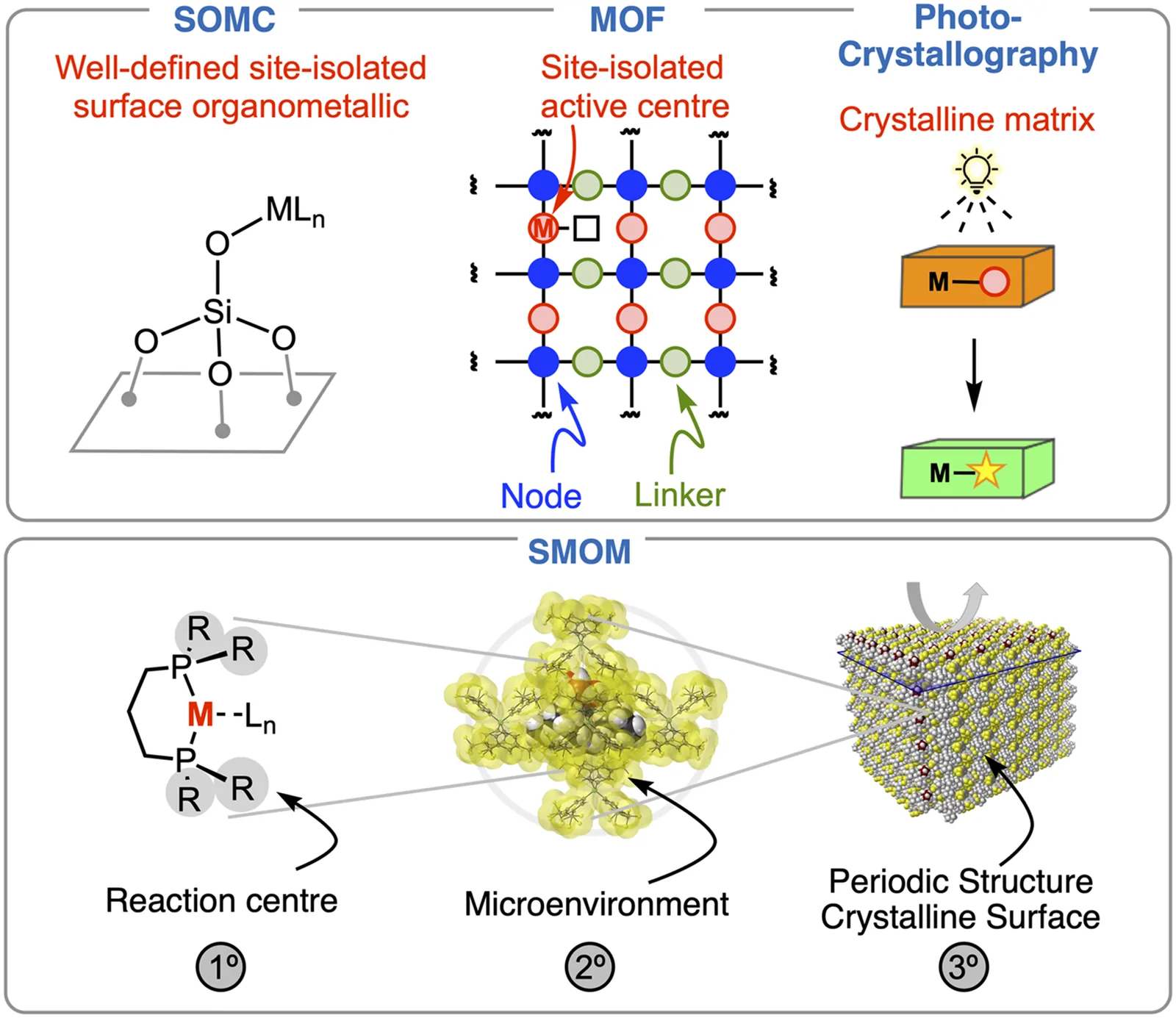
Comparison of SOMC, MOFs, photocrystallography and the SMOM approach to SC–SC reactivity.

Confinement of reactive metal centres to the solid-state, and thus site isolation, removes many of these problems associated with solution-based chemistry. There are a number of methods developed to do this that focus on organometallic materials (Scheme 2).

Surface Organometallic Chemistry (SOMC) provides spatial separation of reactive organometallic centres on platform materials such as silica or alumina, and results in remarkably active catalysts for industrially relevant processes.^12–15^ Characterisation of the surface-bound organometallic species presents a unique challenge compared with molecular-based methods, especially as structural determination using single-crystal X-ray diffraction (SC-XRD) is not possible. Metal Organic Frameworks (MOFs) can have reactive, site-isolated, organometallic centres located in a porous lattice, and have been shown to offer considerable opportunities for synthesis and catalysis, especially in solid/gas processes.^16–21^ However, MOFs rely on the construction of a supporting framework, which potentially limits their more general deployment. Metal-functionalised porous organic polymers offer similar opportunities for catalysis using site-isolated organometallics.^22^

Photochemical transformations of organometallic species in the solid-state provides the opportunity to study light-promoted single-crystal to single-crystal transformations. Reversible processes can also be followed by time-resolved photocrystallography. Such methods provide exquisite, *in situ*, structural characterisation of often short-lived species in a crystalline matrix, at low temperature on a laboratory or beamline diffractometer.^23–31^ However, photochemical activation is less suitable for bulk synthesis, especially due to challenges associated with evenly irradiating a powder (light path effects), which can lead to differences between bulk and surface sites. Interrogation by complementary characterisation methods (*e.g.* solid-state NMR spectroscopy) or onward solid/gas reactivity is also challenging for isolated samples irradiated on the diffractometer. Finally, mechanochemistry has emerged as a powerful synthetic method for solid-state reactions,^32^ but subsequent direct structural analysis of products by SC-XRD can be challenging due the significant reduction in crystal size (comminution^33^), although electron diffraction methods can be used for sub-micron-sized crystals.^34^

These varied approaches to solid-state organometallic chemistry offer unique and exciting opportunities, however, they also each present specific limitations when considering the synthesis, characterisation and onward reactivity of highly reactive organometallic complexes. A complementary approach to solving some of these challenges that is also practical, scalable and deployable in any well-equipped organometallic laboratory, is to use well-defined molecular organometallic crystalline materials for single-crystal to single-crystal (SC–SC) solid/gas reactivity in the bulk. This approach removes the need for solvent, spatially separates reactive metal centres in the crystalline lattice, and potentially allows for the ingress of gaseous reactants and egress of products from the (non-porous) crystalline matrix. While the end point of many synthetic studies is the growth of crystals suitable for structural characterisation using SC-XRD, they now become the *starting point* for solid-state reactivity studies using this methodology.

We have named this approach to synthesis and reactivity *Solid-state Molecular Organometallic Chemistry*, SMOM,^35^ to reflect the conceptual overlap with, but very distinct approach from, surface organometallic chemistry, SOMC, Scheme 2. The concept of solid/gas molecular solid-state organometallic reactivity has been developed sporadically over the last 40 years,^36–40^ notably by Coville,^41^ Siedle,^42,43^ Bianchini,^44,45^ Brammer,^46–49^ and Caulton.^50^ Powers has described general reactivity in the crystalline solid-state as “*in crystallo*” chemistry,^20,24,51^ and we use this terminology in parallel with the specific set of conditions that SMOM offers in combining single-crystal transformation with solid/gas reactivity on synthetically-useful bulk samples.

Single-crystal to single-crystal reactivity offers opportunities to control stability and reaction pathways through additional non-covalent interactions that are brought to bear on, or near, the active metal site through confinement in the crystalline lattice. As Nature shows us with enzymes,^52^ the holistic combination of active primary metal/ligand sites, secondary microenvironment control, and substrate ingress and product egress through the tertiary structure can lead to exquisite control of reactivity.^53,54^ While such concepts are still relatively unexplored using *in crystallo* organometallic chemistry, dynamic single crystals of organic compounds, and coordination complexes, that are adaptive to external stimuli have been extensively studied as materials for potential applications in organic electronics, actuators or catalysis.^55–57^

**Scheme 3:**
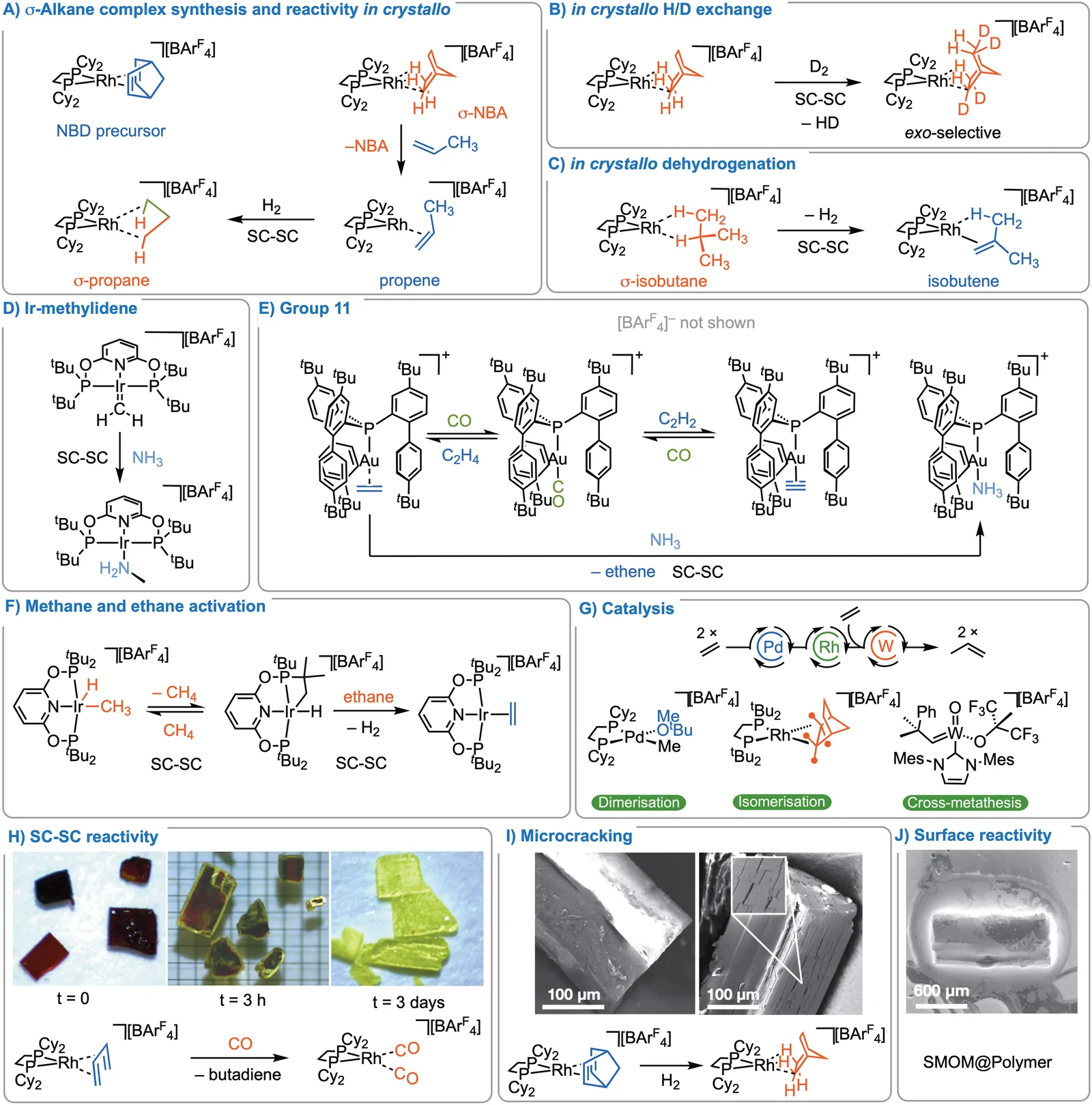
Selected examples of SMOM synthesis and reactivity from our laboratories and examples of physical changes on reactivity. Fig. 1H–J [optical and SEM images] have been reproduced from ref. 65 and 74 with permission from The American Chemical Society, copyright 2015 (H), and the Royal Society of Chemistry, copyright 2020 (I and J), under CC-BY.

Scheme 3 outlines some of the contributions to organometallic chemistry we have made using the SMOM approach. While initially our focus was on the synthesis, characterisation and reactivity *in crystallo* of solution-unstable^58,59^ cationic σ-alkane complexes of rhodium^60–64^ (*e.g.*Scheme 3A ^64,65^), we have extended this approach to other cationic organometallic systems. For example: selective H/D exchange (B),^63,66^ or dehydrogenation (C),^63^ at σ-alkane complexes; an open-shell Co(i) σ-alkane complex;^67^ ligand substitution at group 7 complexes;^68^ Ir–methylidene reactivity with NH_3_ (D);^69^ gold(i)–acetylene or gold(i)–NH_3_ complexes (E);^70,71^ reversible methane activation, or ethane dehydrogenation, at an operationally unsaturated Ir-centre (F),^72^ and catalysis^73–76^ (*e.g.* tandem catalysis in flow conditions, G^77^).

Working *in crystallo* using solid/gas methods and non-porous materials offers a different perspective to solution based chemistry. For example, there is a spatial and temporal component, in that the site of reaction will move from the outside to the interior of the crystal,^73,78^ which can sometimes be observed for slow reactions (*e.g.*Scheme 3H). Microcracking (or shattering) of the crystal may also occur due to resulting lattice strain (*e.g.* as shown by SEM images of single crystals, Scheme 3I).^50,68,74,79^ An example of these spatial elements is the use of the surface of reactive organometallic crystals for catalysis,^76,77^ a visual example being the cationic polymerisation of ethyl vinyl ether at the surface of a crystalline σ-alkane complex to form a SMOM@polymer system (Scheme 3J).^74^

In addition to *in crystallo* photocrystallography and MOF chemistry noted earlier, others have also made important contributions in molecular organometallic, or closely related, SC–SC reactivity. For example ligand substitution and catalysis using,^80^ (NHC)_2_RhCl(N_2_) reactivity with O_2_,^81^ CO_2_ insertion at [(NHC)CuH]_2_,^82^ oxidative addition of C–N bonds at Rh,^83^ reversible ethene uptake at Ag^+^ centres,^84^ reaction of NO and O_2_ at dimeric-Co complexes,^85,86^ activation of H_2_ using Frustrated Lewis Pairs,^87^ thermally induced SC–SC transformation in Lanthanide organometallics,^88^ and aerobic re-oxidation of photoactivated Ti-oxo clusters.^78^

## 2. Scope: a practical guide to SMOM

We are often asked for practical advice on how to proceed with bulk single-crystal to single-crystal solid/gas reactivity. Having developed the method over the last 15 years we now share this advice in this users’-guide to SMOM as a tutorial review. The guide steps though each aspect of SMOM that needs to be considered. Of course, many researchers will be familiar with these methods, and we do not seek to impose on them our approaches, but hope that by bringing them together in one place it serves as a useful reference point. We do hope, however, that by collecting these together, this guide will act to support and inspire researchers to use the method in their own research. There are excellent general articles on photocrystallography,^24,89^ 3D electron diffraction,^90,91^ Surface Supported Organometallic Chemistry^12^ and the use of synchrotron beamlines for *in situ* studies,^92^ that offer complementary methods for organometallic chemistry in the solid-state.

*In crystallo* solid/gas approaches to organometallic chemistry offer potentially straightforward methods for synthesis and catalysis, that add to the toolbox of the practising organometallic chemist. A particular advantage of SMOM is that if no reaction occurs then nothing is lost, and the crystalline material can be reused. Although the objective is often to obtain SC–SC transformations, if crystallinity is lost but the reaction still proceeds productively this outcome may still have benefits compared with the equivalent reaction in solution.^41^ For example the product may be formed more selectively due to the topochemical constraints of the lattice, and there is no requirement for manipulation by subsequent recrystallisation.

## 3. Some design principles for SMOM

**Fig. 1:**
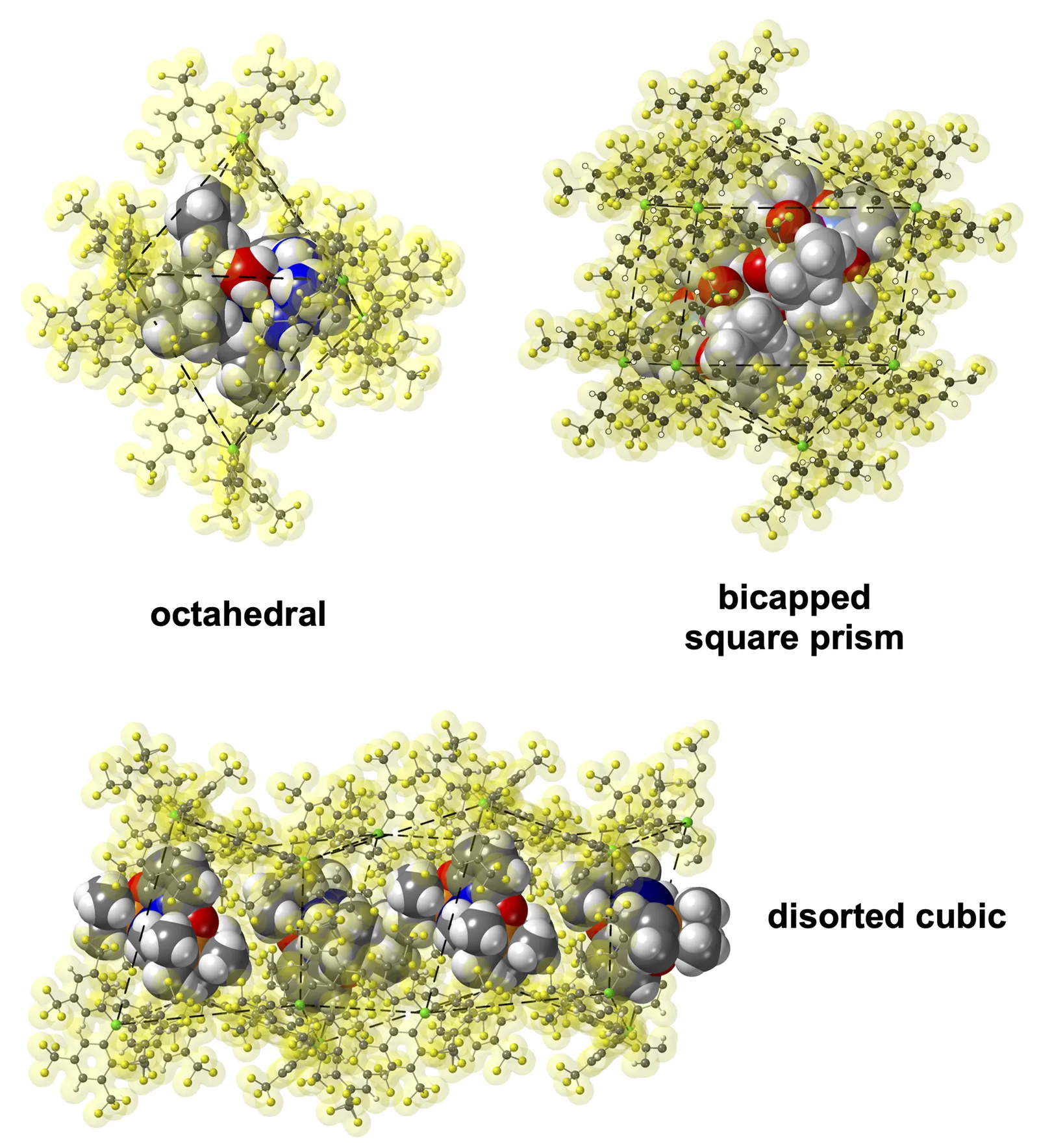
Some common [BAr^F^_4_]^−^ arrangements observed in cationic SMOM systems.

While the confinement of the reactive metal centre in the crystalline lattice leads to the isolation, characterisation, and subsequent reactivity of complexes that are often unstable in solution, there are constraints to this methodology. In order for single-crystallinity to be retained,^79^ in a non-disintegrative^56^ transformation, the change in unit cell volume on reaction is generally below 2%.^37^ There are a few examples where larger changes occur,^68,93,94^ but such disruptions to the lattice may result in crystal shattering through mechanical strain. If long range order is retained in such materials, electron diffraction methods may allow for the structure to be determined when only microcrystalline materials are available.^68,95^ Nevertheless, large structural changes should be avoided, as facilitated by metal ligand/anion design that allows for the reactive volume to be retained. While fluorine-containing groups are useful as they provide lattice plasticity and hydrophobic channels that facilitate ingress and egress of gaseous reactants and products in non-porous crystals,^48,49,80,84,96,97^ examples of systems which undergo SC–SC transformations that do not have fluorous groups are known.^81,82^ In our own work where reactive cations are confined in the lattice, the [BAr^F^_4_]^−^ anion has often been used as a partner. These anions, initially serendipitously and unexpectedly,^60^ provide well-defined nanoreactor^98^ microenvironments that also allow for long-range order to be retained, flexibility at both the reactive site and the arrangement of anions, and channels through the lattice from the –CF_3_ groups. Some of the common arrangements of [BAr^F^_4_]^−^ anions are shown in Fig. 1.

While other anions suitable for SMOM are less well-developed,^42,45,86,99,100^ for example the per-fluorinated aluminates,^101^ we see no reason why they should not support single-crystal to single-crystal reactivity in suitable systems as long as they do not react deleteriously with the cation of interest in the solid-state.^102^ The field is not yet advanced enough to predict which packing arrangements, or indeed anion identities, will work in SMOM. However, that we find different arrangements of the same anion with different metal cations shows that the lattice is – in part – templated by the identity of the cation, and can be influenced by relatively small changes of ligand identity. For example, the isomeric alkene precursors to σ-alkane complexes, [Rh(PCy_2_CH_2_CH_2_PCy_2_)(3-methyl-1,3-pentadiene)][BAr^F^_4_] and [Rh(PCy_2_CH_2_CH_2_PCy_2_)(2,4-hexadiene)][BAr^F^_4_], have ∼O_h_ and bicapped square prism arrangements of [BAr^F^_4_]^−^ anions respectively.^64^ The arrangement of anions can also change during SC–SC transformations, while retaining crystallinity.^68^

While our own work has focussed on cationic systems, there are examples where neutral complexes have been used in solid/gas SC–SC transformations.^80–82^

## 4. Initial considerations

**Fig. 2:**
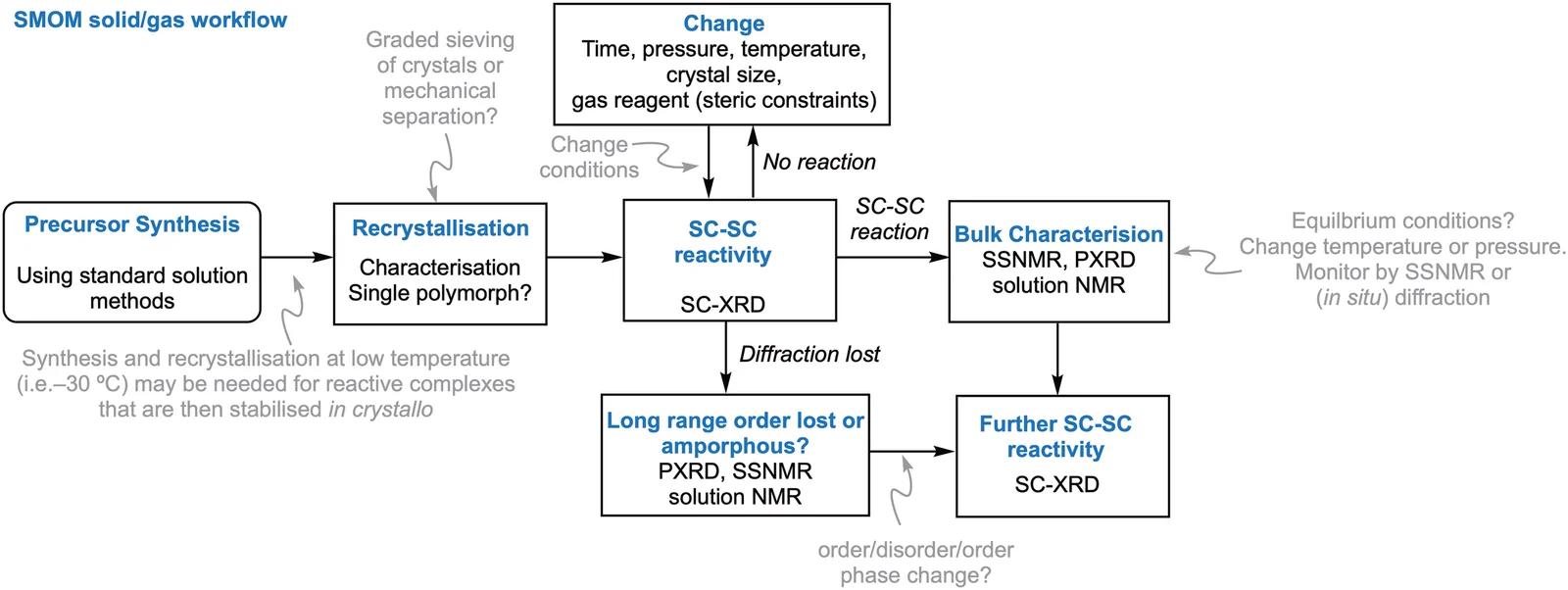
Suggested workflow for *in crystallo* reactivity in SMOM.

When planning for a SC–SC solid/gas experiment on bulk material there are several practical components that ideally need to be in place, consideration of which will help in the full characterisation of any new complexes formed. The workflow diagrammed in Fig. 2 outlines our developed procedures for conducting SC–SC reactions.

(i) The precursor starting material must be isolable in the crystalline phase in good quantities using traditional solution routes, ideally as a single polymorph (as verified by solid-state NMR or powder X-ray diffraction). For analysis by combined single crystal X-ray diffraction, solid-state NMR (SSNMR) spectroscopy and elemental analysis we find 50–100 mg is ideal for a single batch, although of course individual reactions can be run on a much smaller scale than this.

(ii) Good access to a single-crystal X-ray diffractometer is crucial. This allows for crystals to be expediently screened for quality and morphology, before and after reaction. Rapid analysis after addition of a gas allows for analysis of complexes that may only be stable for minutes to hours, even in the crystalline state. Pre-booking (and pre-cooling) the diffractometer before experimental work starts ensures the timely analysis.

(iii) Solid-state NMR spectroscopy is a routine analytical tool, and a particularly important technique for relating the X-ray structure of an, isolated, single-crystal to the bulk composition of the sample.^103^ It is also useful when the material no longer diffracts, either through loss of long-range order or the material becoming amorphous. Variable temperature solid-state NMR also allows for dynamic processes in the solid-state to be probed.

(iv) Access to low temperature solution phase NMR spectroscopy is useful for determining product speciation of highly reactive complexes that only survive low temperature solution conditions. We routinely analyse complexes after SC–SC reactions using solution methods at −90 °C using CD_2_Cl_2_ solvent.

(v) Access to, and training in, the use of gases and the equipment related to gas handling is required if solid/gas reactions are being investigated.

(vi) If crystallinity is lost upon addition of a gas a clean and quantitative chemical reaction may still have occurred. The subsequent recrystallisation of any solution-stable material may enable access to complexes that could not be synthesised cleanly using solution methods.

(vii) Dynamic systems under slow equilibrium can be interrogated by changing pressure, temperature or application of a vacuum. Equilibrium positions are determined by diffraction and NMR spectroscopy.^72^ Those with lower barriers and balanced thermodynamics may require *in situ* methods, such as are available on beam-lines^92^ and as so well-deployed by the MOF community.^17^

## 5. Preparation of crystals

**Fig. 3:**
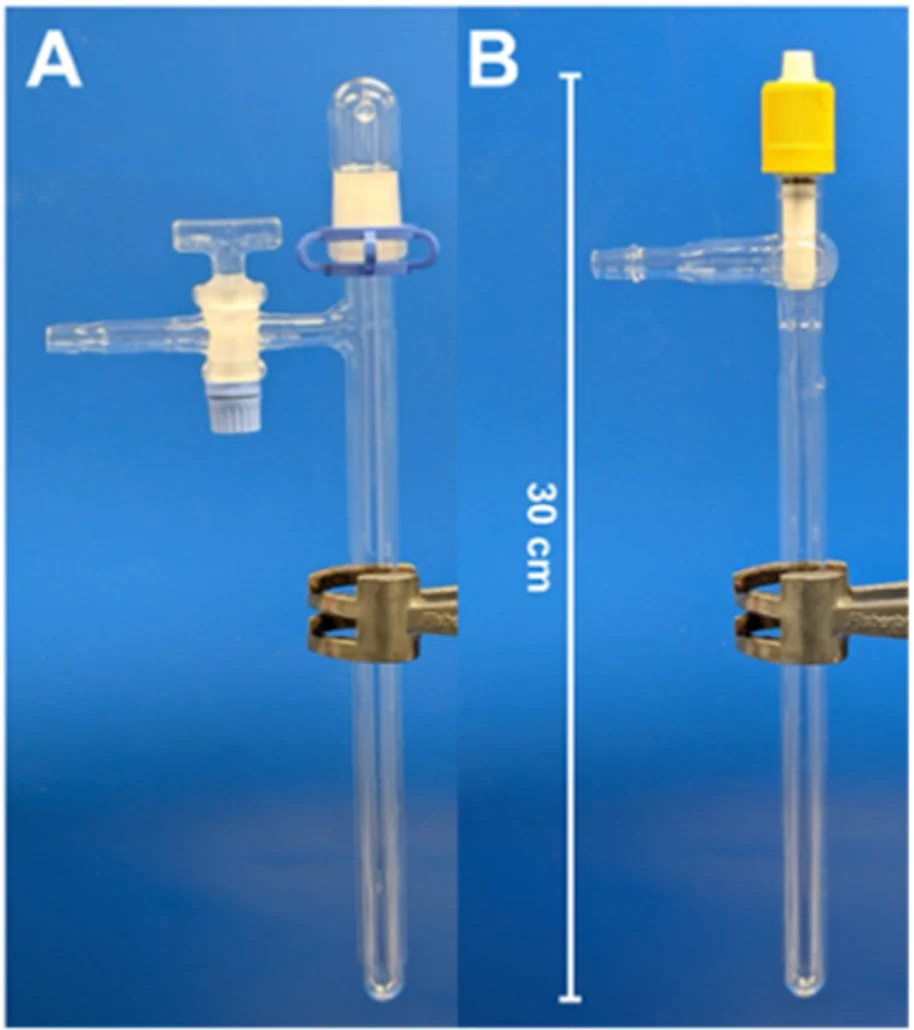
Schlenk (A) and J. Youngs (B) recrystallisation ampoules.

Good quality, crystalline, precursor materials available on the bulk scale (10 mg upwards) are required for SMOM. The use of tall, thin, J. Youngs style ampoules for recrystallisation has been, in our experience, the most effective glassware for small to medium scale recrystallisations (10–100s of mg), and are normally chosen depending on the amount of material to be recrystallised (Fig. 3). A ∼1 : 10 ratio of pro-solvent to anti-solvent is used through layering the anti-solvent on top of the pro-solvent. Recrystallisation of the material then occurs slowly over days encouraging formation of high-quality single crystals. We routinely use dichloromethane or 1,2-difluorobenzene as pro-solvents and pentane, hexane and heptane as anti-solvents.

On occasions for when temperature sensitive recrystallisations of the precursor are required (for example decomposition occurs above −30 °C in solution),^69,72^ they can be carried out by recrystallisation in a glovebox freezer using this glassware, or in a Schlenk-style recrystallisation flask in cases where a glovebox freezer is not suitable or available. The benefits of the J. Youngs style ampoule are that contamination by grease is minimised, and the ampoules – with their threaded pistons – can be cycled into a glovebox when containing atmospheric pressure. These ampoules are less-suited for very low temperature recrystallisations outside of the glove-box environment, as the standard-issue PTFE piston thermally contracts more than the glass ampoule, resulting in the ingress of oxygen and moisture. The stability of some precursor compounds may also not be appropriate with the extended recrystallisation timeframes of a layered recrystallisation. In these cases, rapidly cooling a concentrated solution of the precursor material in an appropriate solvent to −78 °C using a dry ice/acetone bath, and then moving the flask to a −78 °C freezer can yield crops of crystals of sufficient quality for SC–SC reactivity. While all of these methods have been effective for SMOM chemistry in our laboratories, the only requirement is good quality crystals, regardless of the method of recrystallisation ultimately chosen.

Crystals free of co-crystallised solvent molecules in the lattice are generally preferred, as egress can cause loss of crystallinity during SC–SC reactions. However, we have observed retention of crystallinity in cases where solvent in the lattice is retained^70^ or is lost.^68,71^ In cases where *n*-alkanes are found as lattice solvents, methyl cyclohexane can be used as an alternative which has been shown to reduce, or completely remove, residual lattice solvent.

## 6. Single crystal X-ray diffraction

Single-crystal X-ray diffraction is the work-horse analytical technique of SMOM chemistry, and frequent, scheduled, access to a diffractometer is the most important aspect of working within the SMOM space. Such access is important due to the temporal sensitivity of some crystalline reaction products at ambient (or even very low^67^) temperature, that may be relatively short lived. Moreover, it is often necessary to conduct sequential, or repeated, collections to determine the optimal time for the *in crystallo* reaction to achieve full conversion. Complementary analysis by solid-state or solution NMR spectroscopy to determine bulk reactivity are helpful for such optimisation studies.

A routine structure collection of the starting material should always be conducted prior to a SC–SC reaction, regardless of whether the structure is known or not. This allows for a benchmarking of the structure as space group changes may occur during reaction *in crystallo*. It is useful to screen several different crystals of the starting material, to establish fidelity of the bulk material, and if multiple phases, polymorphs,^104,105^ are present then separation should be attempted. This can be by mechanical means, *e.g.* graded sieving or optical selection of crystals, or by recrystallisation from a different solvent mixture. If a polymorphic crystalline material is recalcitrant to these methods, the sample is best treated as an ensemble, and will require multiple data collections on different samples to establish if the transformation of interest is confined to, or differentially affected, by one morphology.^83^ Solid-state NMR spectroscopy combined with solution NMR spectroscopy are useful in confirming polymorphs, as the former may show different environments in the solid-state for each polymorph while the latter will not.^70,72^

Care should be taken with crystals both before and after reaction with a gaseous substrate if the samples are likely to be air sensitive. The frozen oil-drop method provides a barrier to ingress of air and moisture, but of course can only be used after SC–SC reactivity! We have found that most common crystallography oils (*e.g.* Fomblin®) are generally suitable to be used without further purification. In the case of very air-sensitive crystals the crystallography oil may be cycled into a glovebox on a microscope slide, into which the crystals are placed (and thus protected) in the oil, prior to mounting on the diffractometer outside of the glove box.

Typically, very little other preparation of precursor crystals is required before collection of the structure. Noting down the cell parameters can be useful when comparing between pre- and post SC–SC reaction of the crystal. The specific parameters for a collection strategy are unique to each crystal and so will not be covered here. There are, however, some common things to look out for when analysing crystals post SC–SC reaction with a gaseous, or vapour, reactant. It is not uncommon for the crystals to lose some or all long-range order after reaction, and this will be visible as loss or significant degradation of the higher resolution diffraction pattern (Bragg peaks) at high angles. The use of a polariser fitted to a microscope can often be used to visually screen out amorphous material. Even if data can be collected, we often find the resulting solution has decreased in absolute quality (increased *R*-value, mosaicity or crystal cracking^79^). However, this is not always the case, and in certain instances the structure refinement actually improves on SC–SC reactivity.^61^ If crystallinity is lost, or data quality is poor, using smaller crystals (∼150 μm or less) can improve the situation, due to decreased bulk lattice strain and shorter reaction times (higher surface area to volume ratio).

Visual selection of crystalline materials can add bias to the analysis, meaning that the single-crystal analysed may not be representative of the bulk.^106^ As colour changes may not always be obvious during a SC–SC reaction, or can occur with a spatial dimension (*i.e.* surface to bulk),^73,78^ it is always important to complete a full data collection irrespective of the colour of the crystals. Cell parameters often do not change significantly from precursor to product, and thus only a full data collection may reveal if reaction has occurred.

We have found that in some cases loss of diffraction (*i.e.* loss of discrete Bragg peaks) in a SC–SC reaction can be reversed by a subsequent SC–SC reaction with another gaseous reagent in an order/disorder/order reaction.^75,107^ Such order/disorder changes can conveniently be monitored using SSNMR and powder X-ray diffraction, which can show that while diffraction is lost (long range order) local order is retained as signals observed in the SSNMR spectrum remain relatively sharp.^103^

### 3D-ED, synchrotron beamline and single crystal neutron diffraction

Where only very small crystals are available that are not suitable for single-crystal X-ray diffraction on a routine diffractometer SMOM chemistry is still possible, albeit using facilities not available in every department. 3D Electron Diffraction (3D-ED) is a powerful methodology for the structural determination of organometallics where the single-crystalline materials are very small (10s of nm to 5 μm).^90,108^ We,^68,95^ and others,^109^ have used low-flux 3D-ED to study SC–SC transformations where only very small crystalline sizes were available, a result of a microcrystalline precursor or crystal micro-cracking occurring during SC–SC solid/gas reactivity. Further grinding of the crystals is sometimes necessary to achieve the small sizes and plate-like morphology needed. Air-sensitive samples can be loaded onto TEM-grids in a glove-box for transfer to the TEM.

Synchrotron beamlines that have developed *in situ* gas cells^92^ offer exceptional opportunities to study solid–gas SMOM on isolated, single-crystalline samples, as so well demonstrated by the MOF community.^17,110^ Related to photo crystallographic experiments,^24,28,89^ the ability to dose gases under a controlled environment, with precise control of temperature, allows for the characterisation of complexes that are unstable at room temperature after solid/gas reactivity (*e.g.* a propane σ-alkane complex^64^) or to follow reaction progress.^49^ Where suitably large-sized crystals are available single-crystal neutron diffraction is particularly useful for the precise location of hydrides^72^ and/or deuterium atoms arising from H/D exchange,^66^ helped by their different scattering lengths.

## 7. Bulk reactivity considerations

**Fig. 4:**
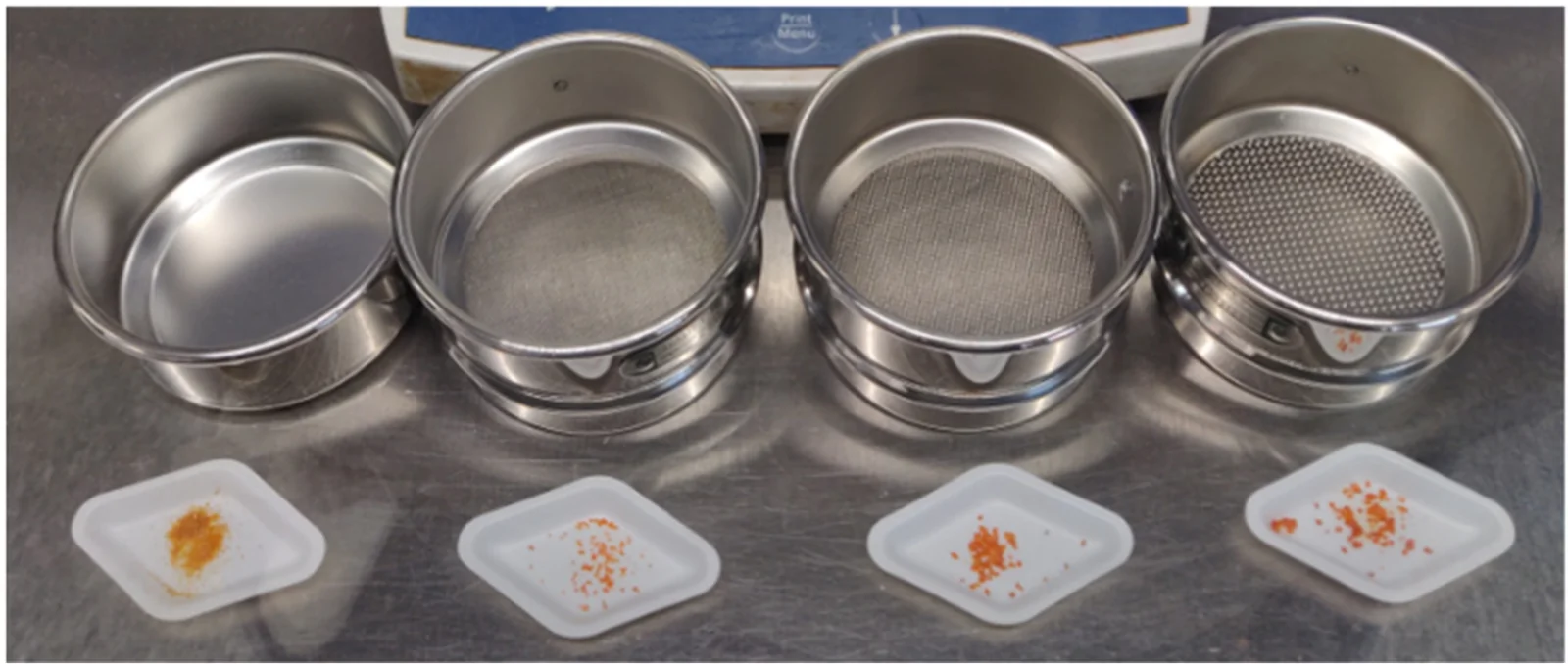
Graded sieving of crystals (from left to right) 0.25, 0.5 and 1 mm sieves.

The intrinsic properties of crystals can have a significant effect on reactivity. For example, rates of reaction can vary for different sized crystals of the same compound, as the diffusion of the substrate gas through the non-porous crystalline lattice will be, to a large extent, controlled by surface area effects. This can lead to incomplete conversion of some crystals, especially larger ones, if present in an ensemble of sizes. A simple solution to this problem is to employ graded sieving of the precursor crystals, a method we have shown to be effective for selecting a specific size-range of crystals, as well as a specific polymorph where it can be grown at a differential size to others, Fig. 4.^72^ This graded sieving provides consistency between analytical samples with regard to the time taken for reaction. Given that we routinely use crystalline material of between 10 and 100 μm for SC-XRD using our in house diffractometers this graded sieving also presents no impediment to structural analysis.

**Fig. 5:**
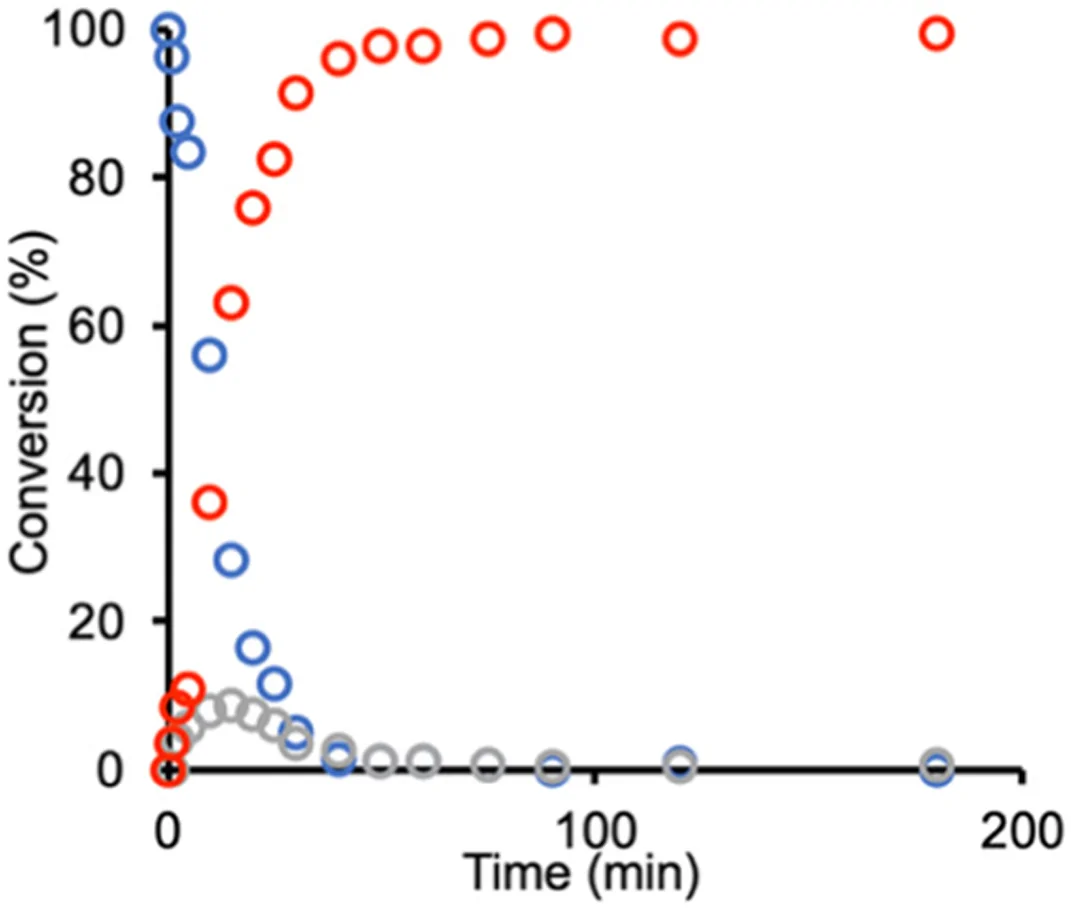
Example of data collected from separate NMR-tube experiments for a SC–SC reaction in the bulk. Each time-point is a separate solid/gas SMOM experiment. Adapted from ref. 106 with permission from The American Chemical Society, copyright 2022, under CC-BY.

A uniform size distribution of crystals is especially important when comparing the temporal profiles of single-crystal solid/gas reactions for kinetic studies. Separate, but identical, sample batches are required for each time point of a reaction, and thus uniform crystal size is ideal. For such bulk studies where SC-XRD is often not required, such as stoichiometric reactivity or catalysis, crystalline material is typically ground in a mortar and pestle, and then separated by graded sieving to produce fine-crystals of cross-section between 71–150 μm.^69,72,75,76,106^ Separate J. Youngs NMR tubes are then pre-weighed in a glovebox, the number of which will depend on the number of data points required for temporal analysis and appropriate repeats. The samples of the relevant ground material are then added directly to the NMR tubes, to limit loss of the powder on weighing boats. Each sample is exposed to the desired reactant gas in the NMR tube for a pre-determined time, before removal under reduced pressure, effectively quenching the reaction. The crystalline samples are then dissolved in deuterated solvent *via* vacuum transfer for analyses by solution NMR spectroscopy, or directly analysed using SSNMR, to determine conversion and thus a temporal profile for the reaction, Fig. 5.

Addition of solvent to these samples will often displace a weakly bound product, such as an alkane. In such cases d_6_-acetone, d_3_-MeCN or fluorinated benzenes^111^ are useful. These solvents may form well-defined adducts in solution with the metal-fragment, releasing the bound ligand of interest that can then be characterised by NMR spectroscopy, mass spectrometry or gas chromatography by vacuum transfer into an additional NMR tube (see section 11). This method has been used, for example, to confirm the formation of alkanes and their dehydrogenated products,^63^ determine products from alkene isomerisation,^77^ and the analysis of deuterium incorporation after H/D exchange reactions.^66,106^

## 8. Gas purity

**Fig. 6:**
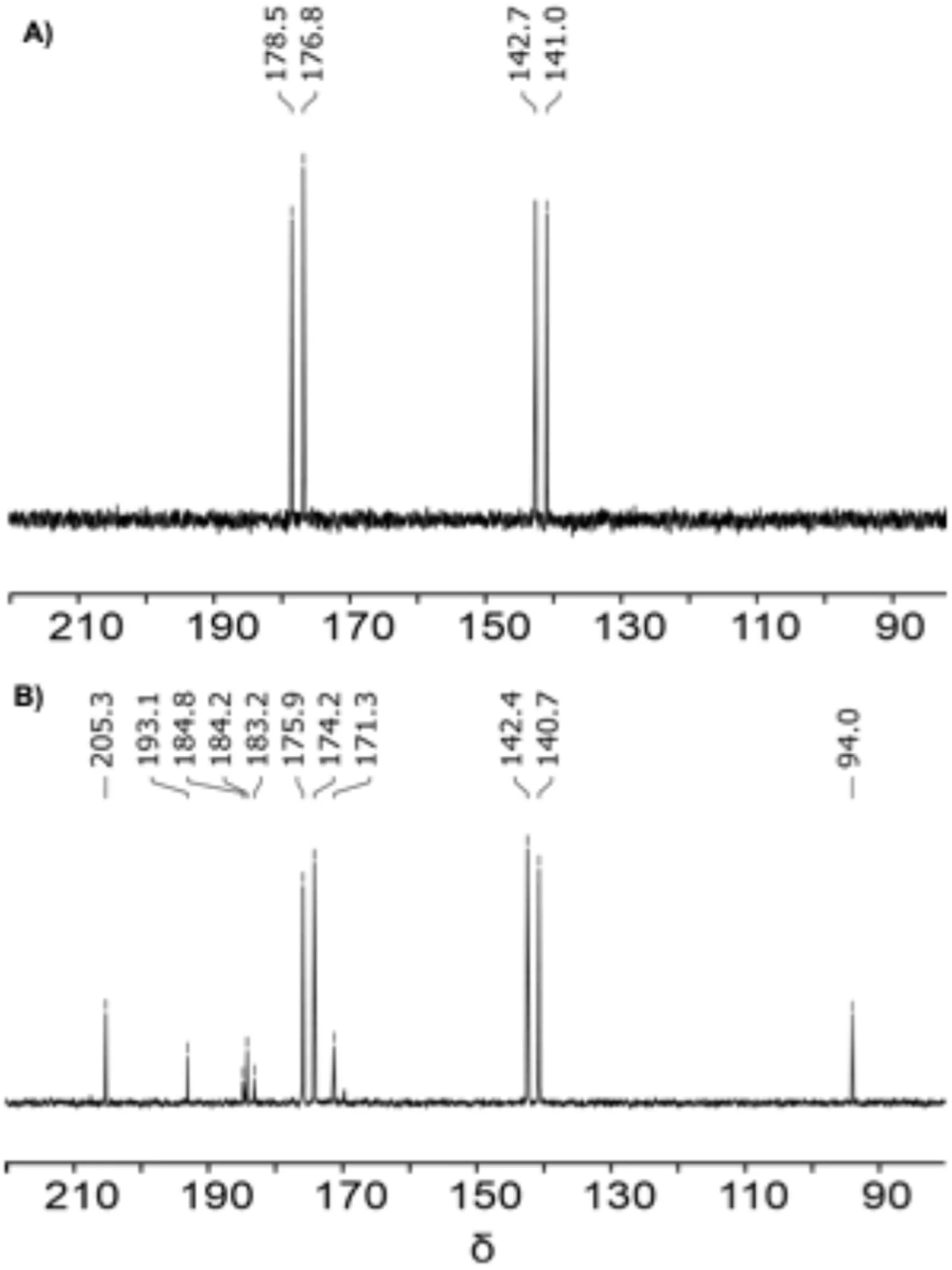
(A) ^31^P{^1^H} NMR spectrum showing pure [Ir(cyclo-^*t*^Bu-PONOP′)H][BAr^F^_4_] after heating [Ir(^*t*^Bu-PONOP)MeH][BAr^F^_4_] under high vacuum at 80 °C. (B) ^31^P{^1^H} NMR spectrum showing compositionally impure [Ir(cyclo-^*t*^Bu-PONOP′)H] after heating to 80 °C under an argon atmosphere for 24 hours.

When performing single-crystal to single-crystal SMOM reactions, the purity of gases used can be critical to obtaining clean transformations. Gas purity of standard house gases such as nitrogen or argon may not be sufficiently high enough to avoid unwanted reactions with trace impurities present (*e.g.* N_2_, H_2_O, O_2_, H_2_). Where appropriate this can be circumvented by performing reactions under vacuum. The example of [Ir(PONOP)MeH][BAr^F^_4_] [PONOP = κ^3^-2,6-(^*t*^Bu_2_PO)_2_C_5_H_3_N] is illustrative, which loses methane on heating single-crystals to 80 °C to form a cyclometallated complex.^72^^31^P{^1^H} solution NMR spectra of the products obtained when this is performed under high vacuum, Fig. 6A, and under argon (99.998% purity), Fig. 6B, show marked differences in purity.

**Fig. 7:**
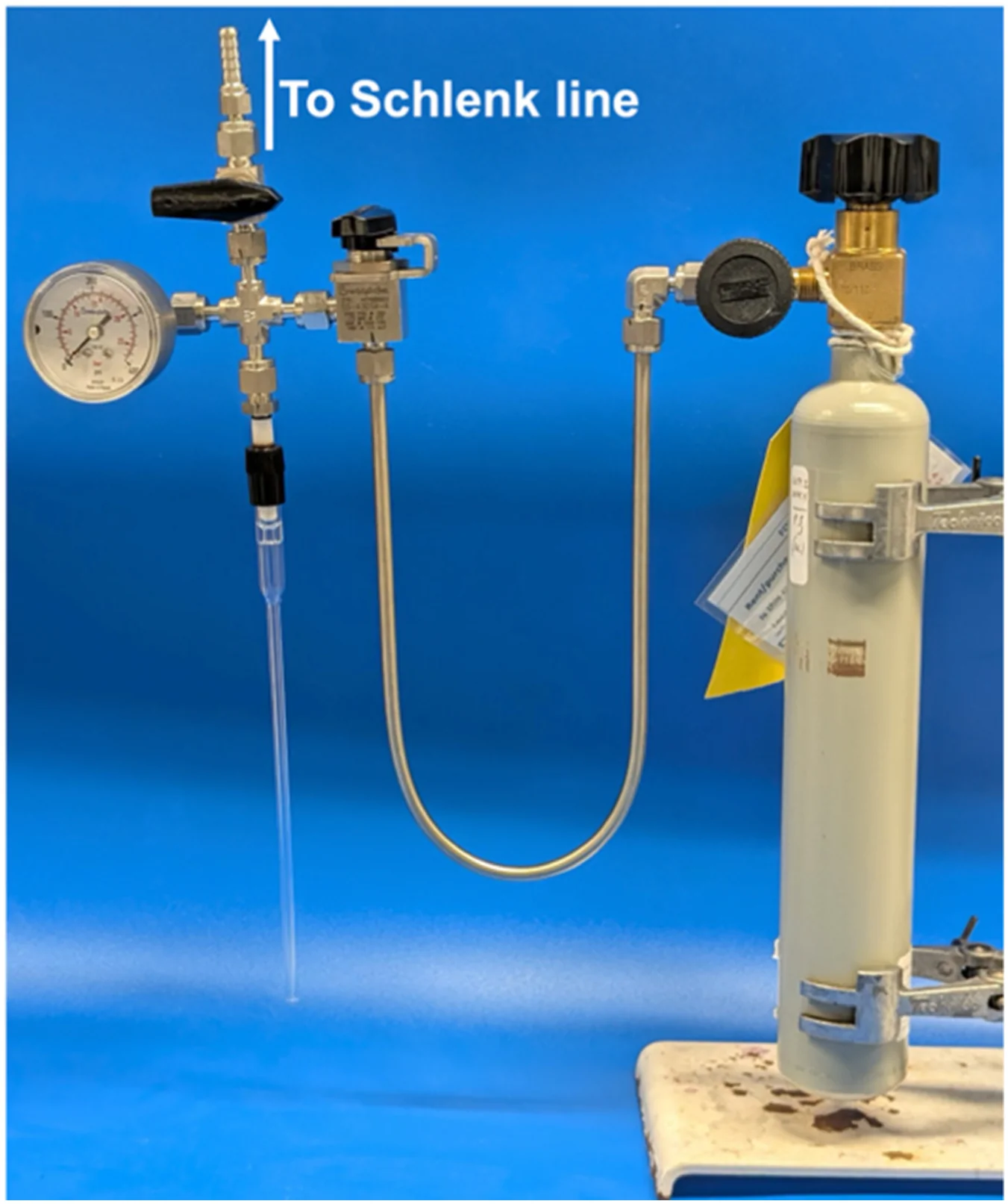
Custom built drying tube packed with Pd/C and 3 Å molecular sieves for removing impurities from CD_4_.

Where vacuum conditions are not possible, for example as a reagent gas needs to be added, purification of house gases is best achieved at the point of use (*i.e.* Schlenk line) by passage through columns packed with activated 3 Å molecular sieves (to remove water) and reduced BASF R3-11G (to remove oxygen). While a drying/catalyst columns directly connected to the Schlenk line is the best solution,^112^ this is not convenient for many ad-hoc SC–SC reactions. An alternative to purifying reagent gases from the common impurities of H_2_O and H_2_, on a reaction scale, is passage through a small stainless-steel column of outgassed, activated, 3 Å molecular sieves and commercially available Pd/C, Fig. 7. This can be interfaced to both a Schlenk line *via* a hose, and the reaction vessel *via* a high pressure threaded connector. We have used such methods to purify CD_4_, that was found to contain hydrogen and water as impurities.^72^ We have also recently reported that commercial D_2_ may also contain trace D_2_O, that can take part in H/D exchange experiments – potentially leading to a misinterpretation of mechanism,^71^ necessitating drying of the gas. “Dry air” can be made by activating 3 Å molecular sieves under vacuum, filling the flask with air and leaving overnight.^78,113^ Removal of ppm-levels of dinitrogen is more difficult however, and can cause problems if dinitrogen adducts are particularly stable.^114^

## 9. Gas handling

**Fig. 8:**
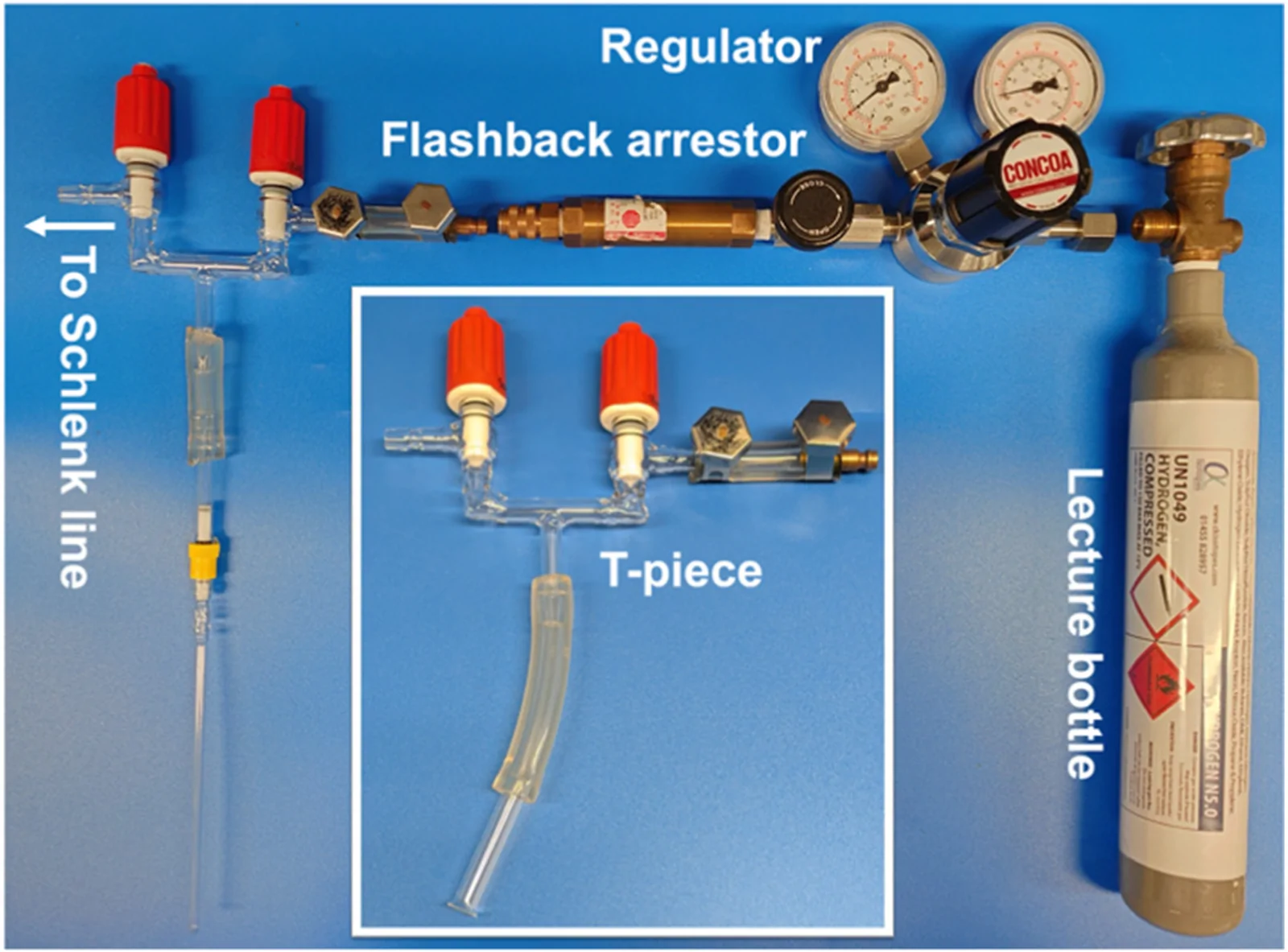
T-piece and two stage regulator/flashback with lecture bottle of hydrogen.

Gas handling carries significant hazards, and suitable training with lecture bottles/cylinders as well as regulators must be undertaken to minimise the risk associated with pressurised, flammable, and/or toxic gas use. Here we describe how we routinely use relatively low-pressure gases (1 to 4 bar absolute) for single-crystal to single-crystal reactions in an air-free manner, but emphasise that local – expert – training is needed before such experiments are conducted. The use of commercially-supplied lecture bottles (∼450 mL) is encouraged, as this reduces the volume of available working gas, and gas storage, to a minimum. In addition, lecture bottles can be conveniently connected to a Schlenk line *via* a custom glass T-piece, which – when used with a flammable gas – is also connected to a regulator equipped with commercially available flashback arrestor, Fig. 8. This setup allows for the entire system to be placed under dynamic vacuum to remove both air and moisture. Once established, the system is then flushed with a minimal amount of the working gas (0.1 to 0.5 bar) and the cycle repeated two more times to bring the whole system under the reactant gas at, *e.g.*, 1 bar. Both ampoules or NMR tubes fitted with PTFE stopcocks (*e.g.* J. Youngs’ style) can be attached *via* vacuum hosing to the T-piece to allow for gas addition to the sample. Before addition of the substrate gas, the reaction vessel is evacuated to a suitable pressure (*e.g.* 5 × 10^−2^ mbar) and then back filled with the substrate gas.

**Fig. 9:**
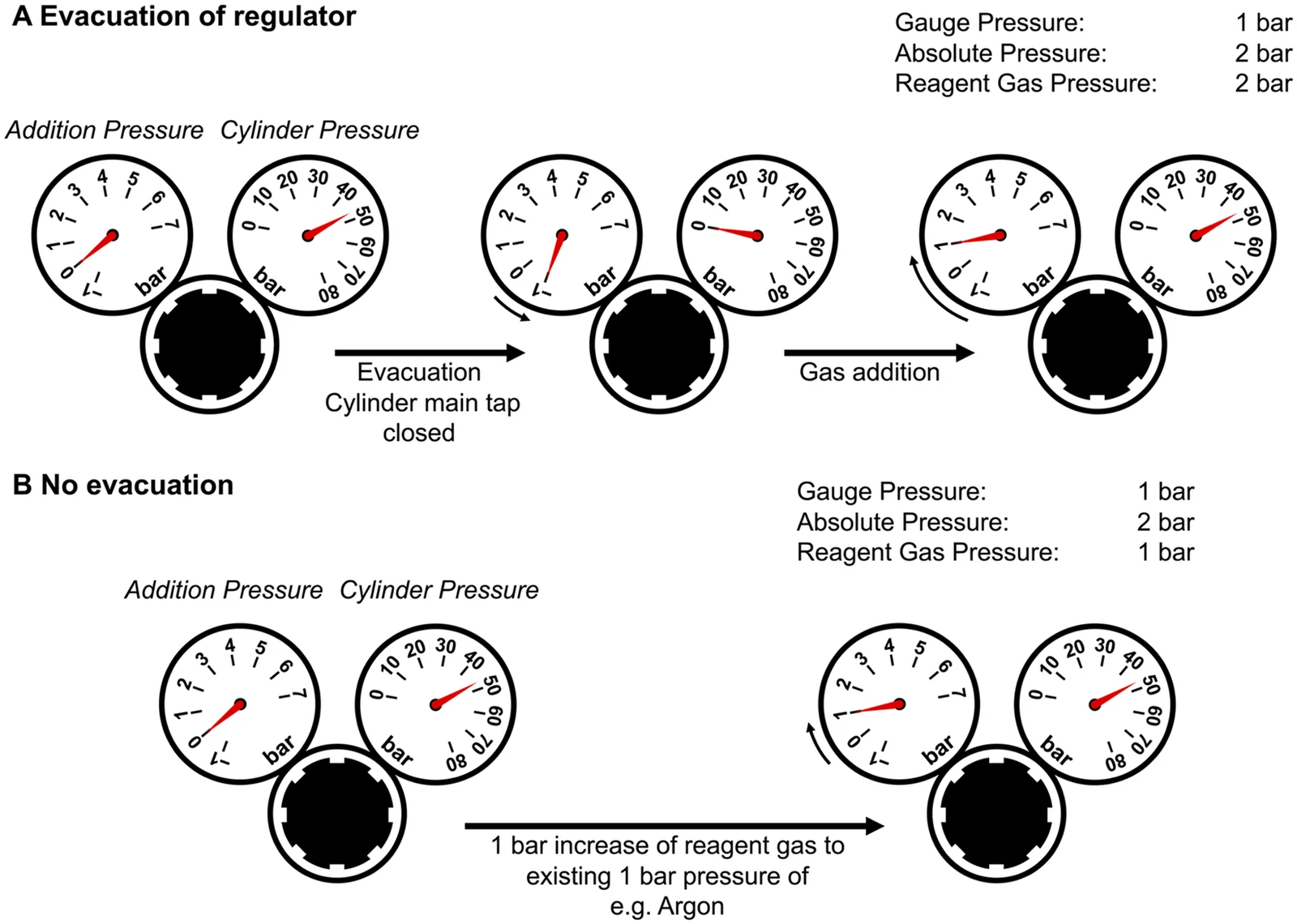
Two stage regulator gauges showing the differences between *bar gauge* and *bar absolute* under different filling regimes. (A) Evacuation of the regulator prior to gas addition, (B) No evacuation.

It is important to note that two sets of terminology can be used when referring to the pressure of a static system: *gauge* and *absolute* pressure. While both are valid measurements, they can lead to different pressures of the substrate gas relative to a reaction vessel under vacuum. Fig. 9A exemplifies this. Evacuation of the regulator to the closed cylinder head leads to a measured pressure of −1 bar. Subsequent filling from the cylinder to a gauge pressure of 1 bar gauge leads to 2 bar absolute. Further confusion is introduced if the regulator head is not evacuated (Fig. 9B), as 1 bar gauge will reflect 1 bar cylinder gas plus the residual gas in the line. Using the *bar absolute* terminology is thus unambiguous. An example is where a reading of 1 bar gauge is actually 2 bar absolute when a reaction vessel is filled from being under vacuum.^115^

### Addition of multiple gases

Many of the techniques described below are complementary to traditional vacuum line techniques, which rely on manometry using a u-tube mercury manometer. For example, condensable gases may be measured into evacuated volumes and non-condensable gases cryogenically adsorbed onto degassed molecular sieves or silica.^112^ Many laboratories do not have access to these specialised vacuum lines, especially the required mercury manometers which are being phased out due to exposure concerns. The following gas handling procedures can be employed to achieve a gas mixture of two or more gases which can then be added to crystalline material for single-crystal to single-crystal reactions.

### In cases where gases are condensable

Mixtures of gases can be made up prior to addition to crystalline samples. For example, two condensable gases are added to identical but separate vessels (Youngs NMR tube, ampoule *etc.*) at half the desired final pressure at room temperature. One gas is then condensed onto the second under a static vacuum using a cryogen such as liquid nitrogen. It is especially helpful to have the lower boiling gas in the receiving flask when transferring under a static vacuum. This mixture can then be added *via* condensation to crystalline samples. It is important that all the vessels are of the approximate same volume as to maintain a constant and safe working pressure throughout. It is also important to remember that liquified gases have very large expansion ratios (*e.g.*, ethylene = 1 : 482) so caution must be taken to not condense large amounts of gases into small volumes, as these pose a risk of boiling liquid expanding to an unexpectedly high-pressure on warming. Lusac's law (*P*_1_/*T*_1_ = *P*_2_/*T*_2_) should be used to calculate the total pressure of a system at room temperature. Blast shields and appropriate personal protective equipment, *e.g.*, leather gloves and face shields, should also be used. Secondary containment vessels preferably made of hard plastic or metal are also advised when working with condensable gases, in case of explosion/implosion.

### Where one of two gases is not condensable *i.e.*, hydrogen

The boiling point of H_2_ is −252.9 °C and thus it will not condense when using liquid nitrogen as a cryogen. H_2_ must therefore be added last when adding both a condensable gas and hydrogen together.^116^ For a 1 : 1 mixture, the condensable gas can be added directly to the sample vessel at room temperature at half the final target pressure. Under a closed system the sample vessel is then cooled using liquid nitrogen until the substrate gas condenses/freezes. H_2_ is then directly added to the cooled vessel at half the desired final pressure of the two gases, taking into account the expansion of the gas on warming to room temperature (*P*_1_/*T*_1_ = *P*_2_/*T*_2_). The vessel is then warmed to room temperature to reach the required pressure.

### Where both or multiple gases are non-condensable

Mass-flow controllers or specialised gas mixing equipment are used to achieve the desired pressure and mixtures of substrate gases.

### Higher pressure experiments

**Fig. 10:**
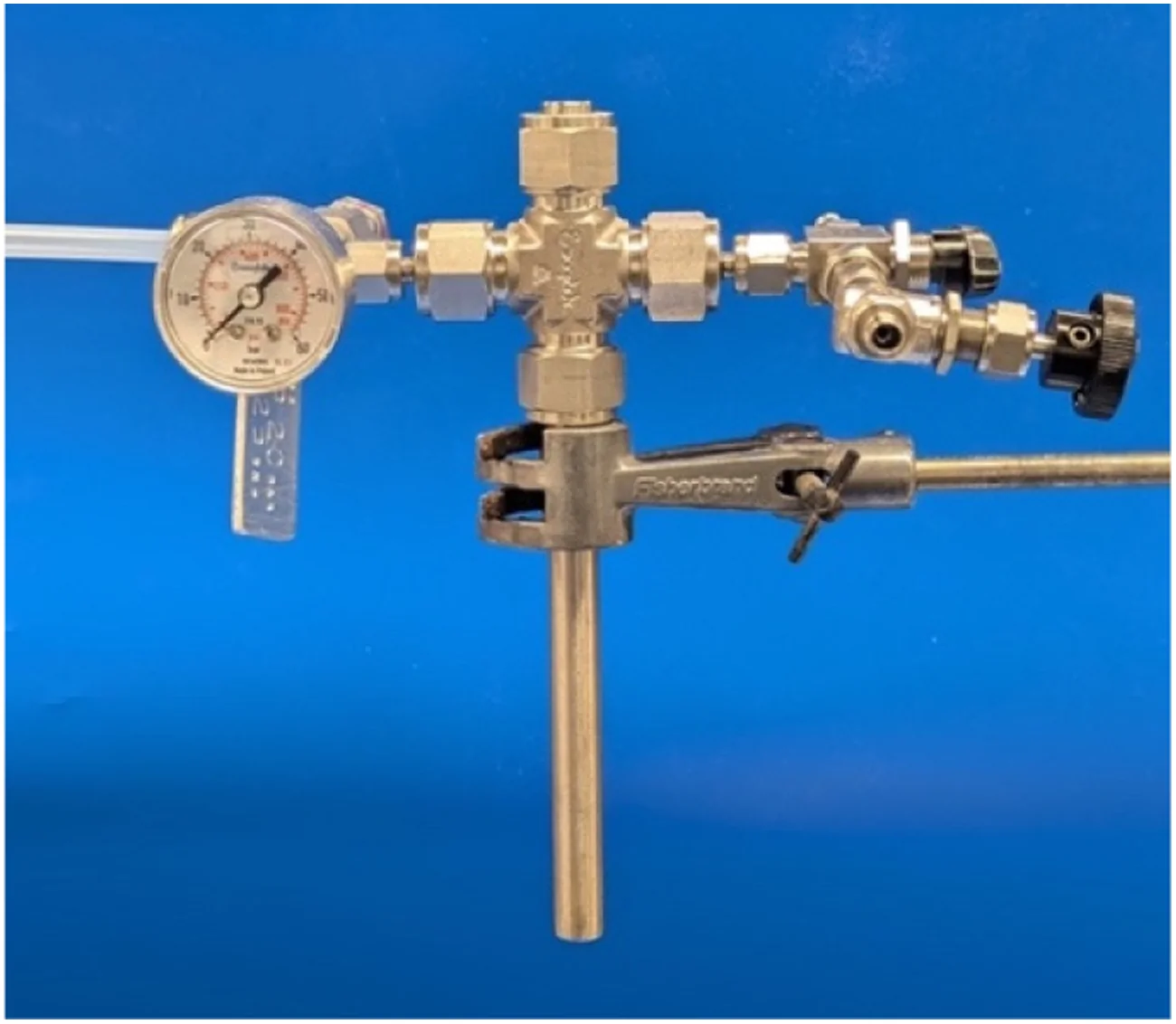
Custom made pressure reactor allowing for SC–SC reactions up to 20 bar working pressure.

We routinely use heavy walled high-pressure borosilicate glass NMR tubes^117^ as these also allow for gas-phase NMR spectroscopy to be carried out during the course of a SC–SC synthesis or catalysis. Alternatively, high pressure reactors are widely available and come in various shapes and sizes. While these can be useful for carrying out larger scale reactions, they are not cheap. We have used a custom pressure vessel (Fig. 10) which has been designed to allow for the reactor to be interfaced with most gas cylinders, as well as a Schlenk line.^118^ This reactor fits into the large antechamber of a standard glovebox. Furthermore, its long downward tube may be placed into a cooling/cryogenic bath to condense gases to allow for a higher room temperature pressure. The reactor should be hydrostatically pressure tested to give definitive safe working limits. An example is a SC–SC reaction under 20 bar of a non-flammable gas at 80 °C. The reactor was taken into the glovebox, crystals were placed into in a borosilicate glass NMR tube then placed within the reactor and sealed. The reactor was interfaced with the Schlenk line and gas-cylinder, placed behind a blast shield, and pressurised to 20 bar directly from the cylinder. Following the attempted SC–SC reaction, the reactor was attached to a Schlenk line, vented using the needle-valve attachment and taken back into a glovebox to retrieve the crystals for analysis.

## 10. Solid/vapour reactions

**Fig. 11:**
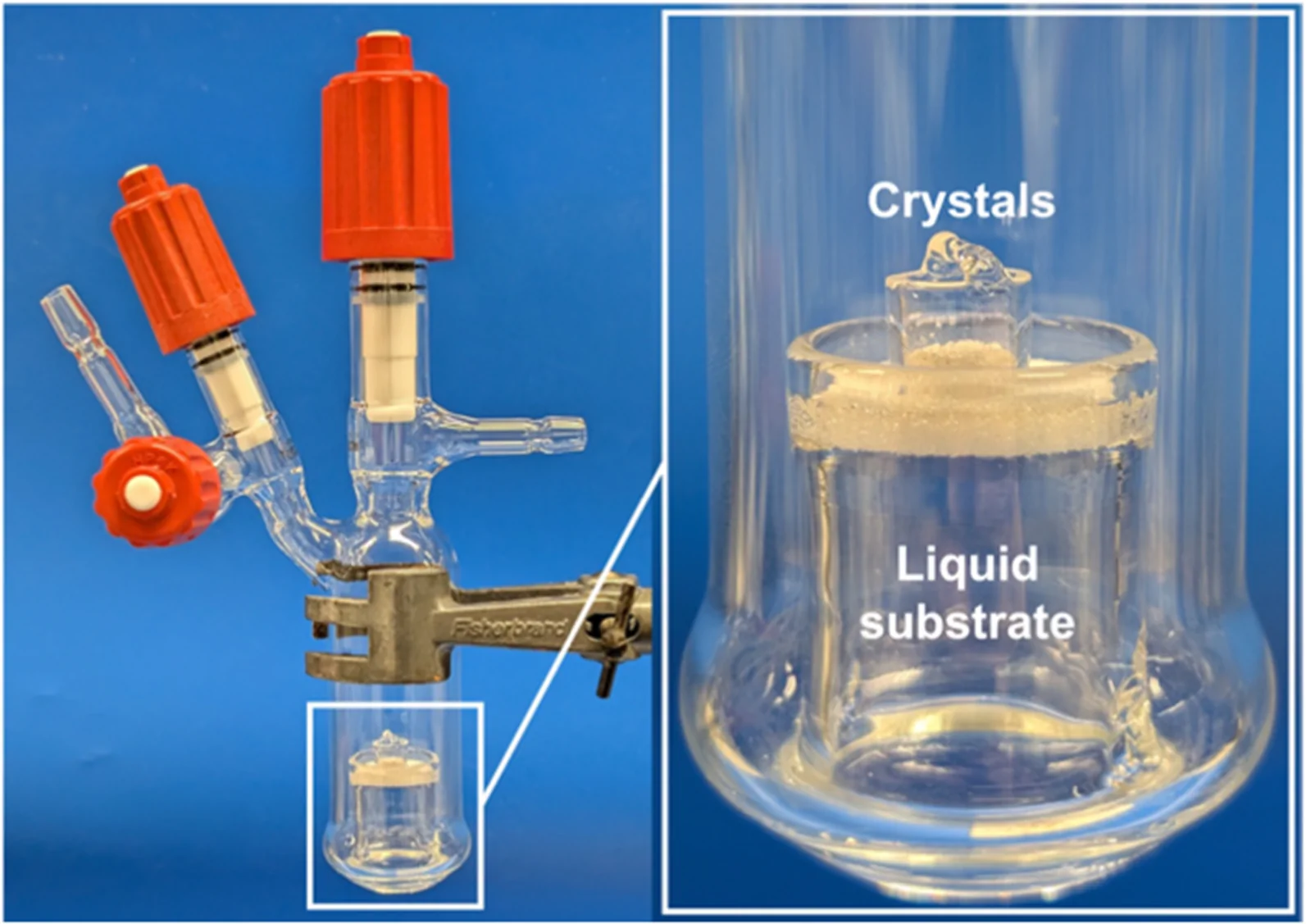
Ampoule design and fritted boats for liquid vapour diffusion SC–SC reactions. Note there is also a hole in the frit support to allow for liquid movement.

SC–SC reactions of air-sensitive compounds with volatile liquid reagents can also be carried out, if contact between the liquid and crystals is avoided (assuming the crystal may dissolve in the liquid). While a number of methods for this exist,^39,47,48,119^ we have designed a custom built reaction vessel consisting of a fritted stage that supports glass crucibles which hold the crystals. These crucibles also have a fritted base to allow for the diffusion of liquid vapours to the sample avoiding pooling of liquids around the crystals and a handle that allows them to be lifted in and out of the flask using a metal hook (Fig. 11). Typically, working inside a glove box, the crystalline sample is placed in the crucible and lowered onto the fritted stage within the reaction vessel. Outside of the glove box, the reaction vessel is then connected to a Schlenk line so that the desired liquid can be added to the bottom of the flask, *via* syringe. A partial vacuum is applied to generate a higher vapour pressure of the liquid. Following the SC–SC reaction, the liquid can be carefully removed using a cannula, avoiding the crucible, then the flask is placed under vacuum to remove any traces of liquid. Subsequently, the flask can then be taken into a glovebox to retrieve the crucible using the metal hook for subsequent analysis of the crystals by SC-XRD or NMR spectroscopy.

**Fig. 12:**
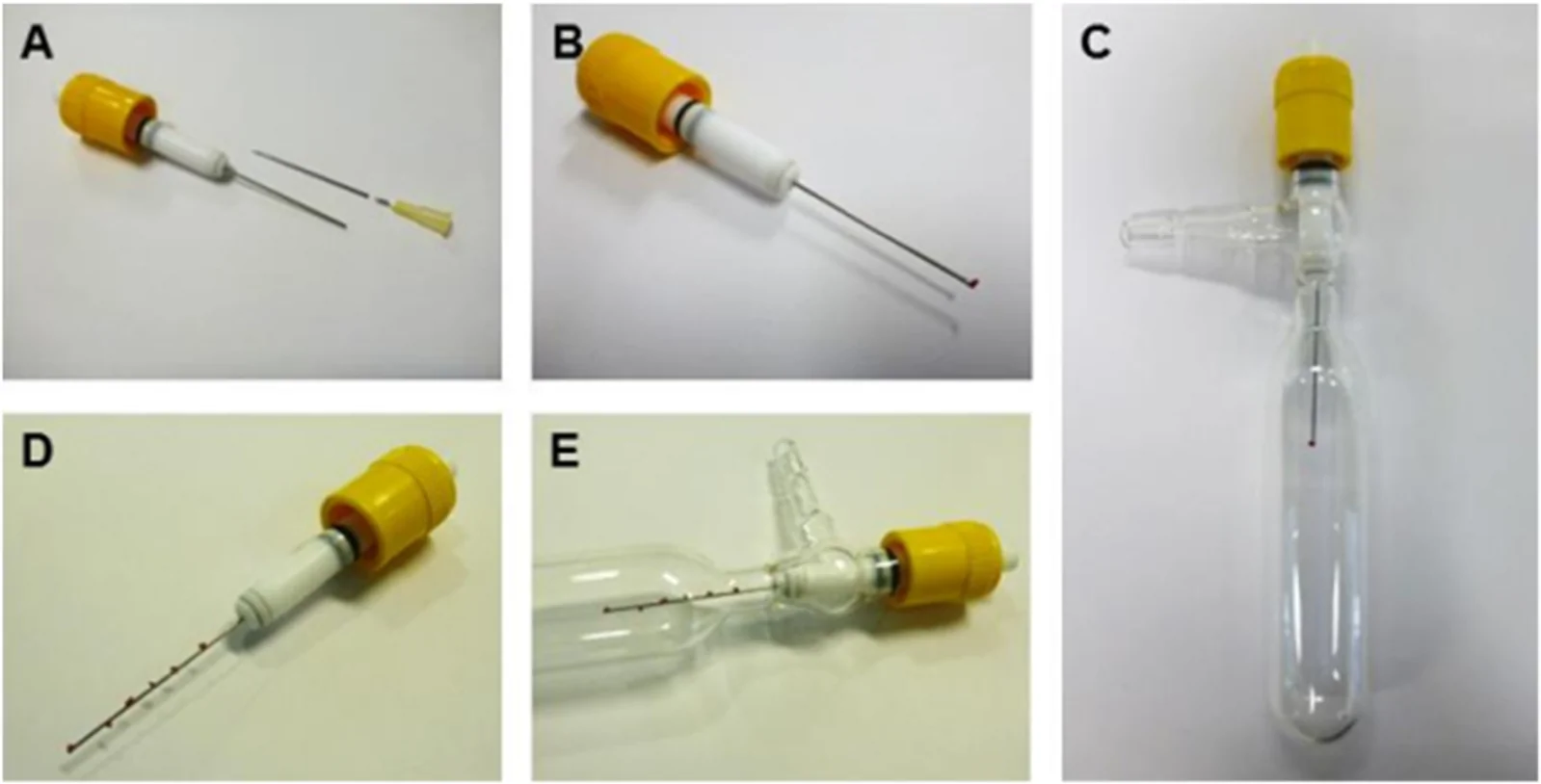
Modified J. Youngs tap with pressed cut needle with attached single crystals for liquid vapour reactions showing the stages of preparatin (A) to (E). Reproduced from ref. 73 with permission from the Royal Society of Chemistry, copyright 2020, under CC-BY.

An alternative option for solid/vapour reactions is to press a cut needle into the base of a PTFE piston. Crystals can then be mounted to the length of the needle using a small amount of silicon grease and inserted into the ampoule (Fig. 12). Volatile liquids can then be carefully condensed *via* vacuum transfer into the flask. We have shown this to be a practical method for the polymerisation of ethyl vinyl ether on the surface of single-crystals of a reactive σ-alkane complex.^74^

## 11. NMR spectroscopy in SMOM

### Low temperature solution NMR spectroscopy

**Fig. 13:**
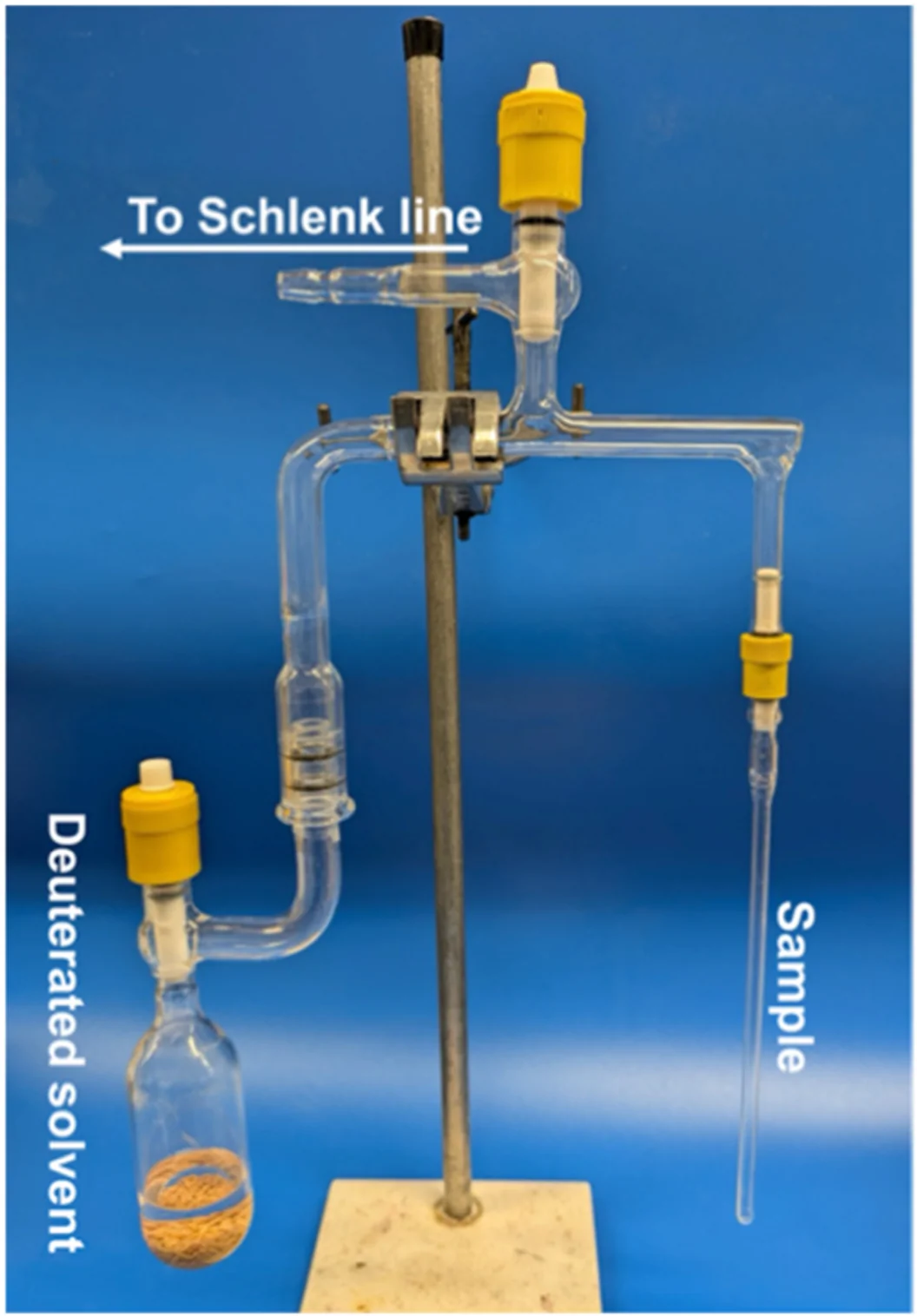
Vacuum transfer apparatus for condensation of deuterated solvents onto crystalline solid-samples. The NMR tube containing the sample is placed inside a dewar containing a suitable cryogen.

Solution NMR spectroscopy allows for detailed characterisation data of the reaction products from solid/gas SMOM to be collected, and low temperatures, and relatively non-coordinating solvents such as CD_2_Cl_2_, are best used. Low temperature NMR analysis is conducted by removal of the gaseous substrate from the SC–SC reaction, followed by addition of CD_2_Cl_2_*via* vacuum transfer onto the solid crystalline samples using a suitable cryogen to condense the solvent, usually liquid nitrogen, Fig. 13. Solvent can be transferred from a larger ampule (shown) or a pre-measured volume from another NMR tube. The NMR tube containing the sample can then be stored at the desired low temperature, or frozen in liquid nitrogen, prior to measurement by NMR spectroscopy. To prevent solvent thaw-induced cracking of the glassware, the sample tube is carefully thawed using a brief acetone wash, then rapidly transferred to a dry ice/acetone bath. The sample is then placed into a pre-cooled NMR spectrometer, and held at the desired temperature for five minutes to equilibrate, usually during shimming, tuning and matching routines.

### Solid-state NMR spectroscopy

Solid-state NMR spectroscopy, especially of ^13^C and ^31^P nuclei, is a powerful analytical technique.^103,120^ Analysis of SC–SC reactions using SSNMR techniques is conducted by first placing the sample under vacuum to remove any traces of the reactant gas. The crystalline material is then transferred into a glove box and crushed into a microcrystalline powder using a pestle and mortar. The samples are then packed into a zirconia solid-state NMR rotor, also inside a glovebox, before capping and transferring to the spectrometer. Transfer of the rotor from the glovebox to the spectrometer is best conducted with the rotor in an argon filled sample vial to avoid ingress of air. We have found that even very air-sensitive samples, such as σ-alkane complexes, can be analysed without significant decomposition using this method. Unpacking of samples from rotors can be performed inside the glovebox, after cycling the rotor in *via* the antechamber.

**Fig. 14:**
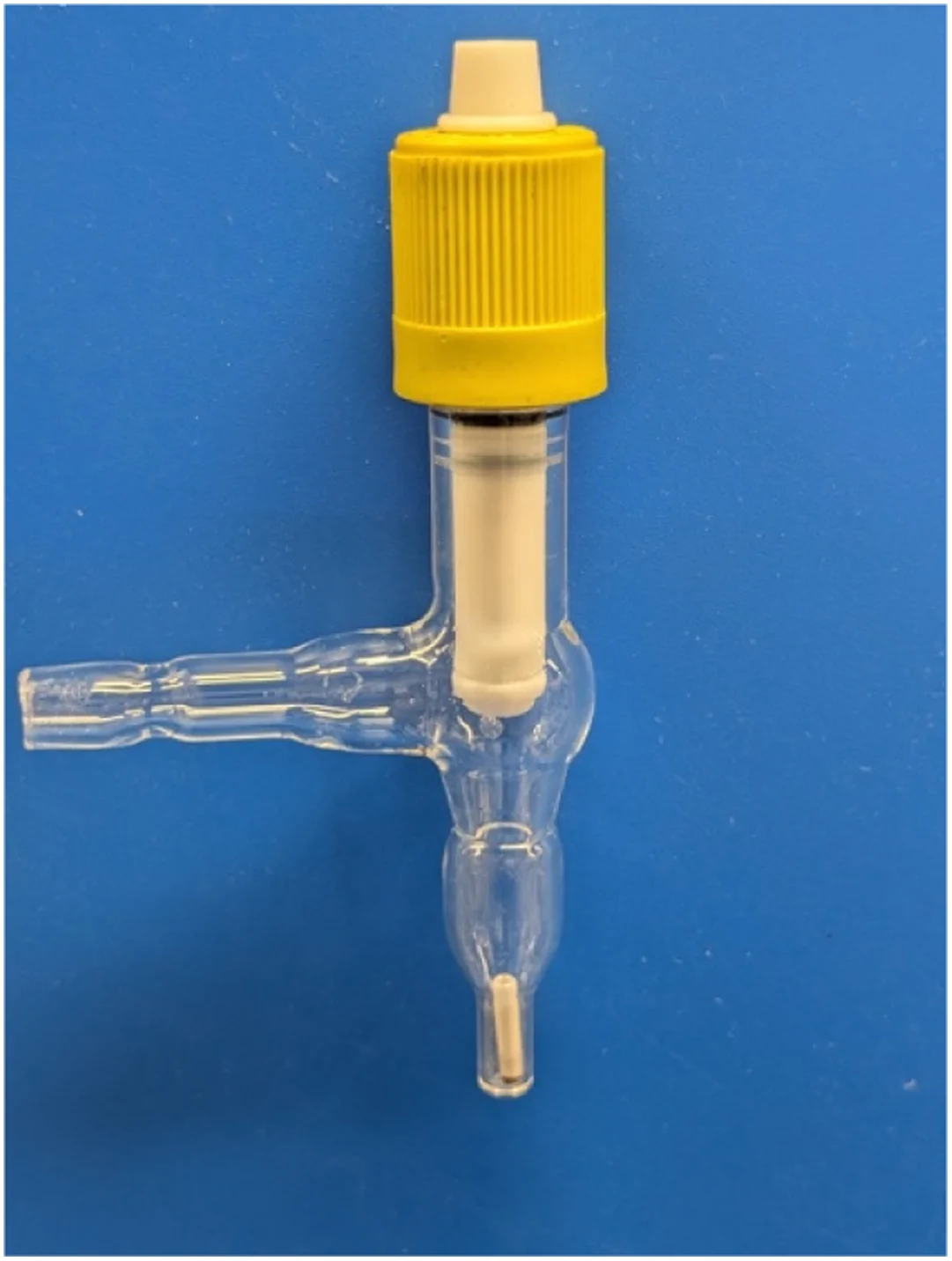
Customised J. Youngs ampoule for addition of gaseous substrates to SSNMR rotors.

*In situ* solid-state NMR analysis can also be carried out using a modified J. Youngs ampoule custom built to fit a SSNMR rotor, Fig. 14. A finely ground powder of precursor microcrystalline material is placed into a zirconia solid-state NMR rotor and placed into the custom J. Youngs ampoule. The reaction is then carried out under a pressure of the reactive gas. At a selected time point the PTFE tap is removed and the rotor is loosely capped under a flow of gas. The rotor is then transferred to an Ar-filled glovebox to fully cap the rotor, before transferring to the spectrometer. While this technique is limited by the slower diffusion of the reagent gas through the path length of the sample in the densely packed rotor, and the pressure of gas sealed in the rotor will be limited to just over atmospheric, it is particularly useful for straightforward reactions where there is no temporal constraint.

### Gas phase NMR analysis

While gas phase NMR spectroscopy has been known for some time,^119,121^ and the use of ^129^Xe NMR spectroscopy to examine porous structures is well known,^122^ it is generally an underutilised technique for practitioners of organometallic chemistry. We have found gas phase NMR spectroscopy to be a useful technique for interrogating solid/gas reactivity to determine reaction outcomes, particularly ^1^H and ^2^H NMR spectroscopy. Reaction profiles can be obtained from the relative integrals of substrates and product gas phase signals, allowing for kinetic studies.^73,77,123–125^ Modern NMR spectrometers increasingly rely on automated locking and shimming routines that cope poorly, at best, with these types of experiment. Shimming directly from the FID is preferred (often technically described in the literature as “shimmed off-lock to maximise the FID area”) and is best taught practically by local expertise. With good manual shimming, reasonably sharp, well-resolved signals can be obtained. Due to this lack of lock when running gas phase NMR studies, overall timescales should be kept relatively short to avoid field drift and the attendant distortion of peaks. Peak widths at half height <4 Hz are generally achievable, meaning multiplet structure is often resolvable. ^1^H *T*_1_ times are typically short in gas phase NMR (although *T*_1_ times can be related to the pressure of the gas^126^). Ideally, this allows for quantitative data to be rapidly obtained using a 90° pulse, and short inter pulse delays, using relatively low pressures of gas. For slower reactions a single 90° pulse followed by a delay of minutes between the next scan can be employed. This method allows for the *T*_1_ time of different gases to be mitigated against, and accurate relative integrals can be achieved of mixtures of gases. The internal diameter of the NMR tube will also affect diffusion (mixing) and needs to be considered when comparing line widths or relative integrals between different samples. We,^76^ and others^121^ have also used gas phase NMR spectroscopy to follow solid/gas catalysis using *para*-hydrogen induced polarisation techniques. A useful summary of gas phase NMR techniques has been published.^124^

### Gas chromatography

Direct sampling of reaction vessels by gas chromatography is possible, that provides additional opportunities for analysis of the headspace. This can be achieved by directly interfacing the reaction vessel to a suitable gas chromograph equipped with a gas-sampling loop. Alternatively, by interfacing a J. Youngs NMR tube to the reaction vessel (*e.g. via* a T-piece) allows for the gas to be collected, and then injected to the GC using a gas-sampling loop.

## 12. Conclusions

*In crystallo* solid/gas solid-state molecular organometallic (SMOM) chemistry offers unique opportunities in organometallic synthesis and catalysis. The methodology provides straightforward to implement methods to synthesise complexes that are challenging to isolate and characterise using solution routes. Whether this is an exotic complex that is fleeting – at best – in solution,^60,67^ the preparation of a new complex cleanly and quantitively using solid/gas methods,^70,72^ or a pre-catalyst (or resting state) for an efficient catalytic process^75,77^ is up to the researcher. The power of the method comes in its simplicity, combined with the variety of analytical techniques that can be used to characterise the materials. The confined lattice environment offered *in crystallo* provides additional opportunities to control reaction pathways and selectivities in both synthesis and catalysis, not available using solution routes.

The SMOM approach adds to the toolbox of methods that the chemist can use exert precision control over reaction outcomes, and is wholly complementary with other approaches towards molecular confinement such as photocrystallography,^24^ MOF materials,^17^ mechanochemistry^32^ and SOMC.^12^ We hope that this tutorial review is a useful addition to the field, and that you find success in using these methods and approaches in your own chemistry.

## Author contributions

KA assembled and coordinated the first draft. ASW wrote the final draft. All Authors contributed to the writing of manuscript.

## Conflicts of interest

There are no conflicts to declare.

## Data Availability

No primary research results have been included and no new data were generated or analysed as part of this review.
